# Evaluation of Alternative Lithium Salts for High‐Voltage Lithium Ion Batteries: Higher Relevance of Plated Li Morphology Than the Amount of Electrode Crosstalk

**DOI:** 10.1002/smll.202410762

**Published:** 2024-12-15

**Authors:** Anindityo Arifiadi, Lennart Wichmann, Tobias Brake, Christian Lechtenfeld, Julius Buchmann, Feleke Demelash, Peng Yan, Gunther Brunklaus, Isidora Cekic‐Laskovic, Simon Wiemers‐Meyer, Martin Winter, Johannes Kasnatscheew

**Affiliations:** ^1^ MEET Battery Research Center Institute of Physical Chemistry University of Münster Corrensstr. 46 48149 Münster Germany; ^2^ International Graduate School for Battery Chemistry, Characterization, Analysis, Recycling and Application (BACCARA) University of Münster Corrensstr. 40 48149 Münster Germany; ^3^ Helmholtz Institute Münster IMD‐4 Forschungszentrum Jülich GmbH Corrensstr. 46 48149 Münster Germany

**Keywords:** cross‐talk, full‐cell, high surface area lithium (HSAL) plating, high‐voltage operation, lithium battery, lithium conducting salts, lithium ion battery, rollover failure, transition metal dissolution

## Abstract

Increasing the upper cut‐off voltage (UCV) enhances the specific energy of Li‐ion batteries (LIBs), but is accompanied by higher capacity fade as a result of electrode cross‐talk, i.e., transition metals (TM) dissolution from cathode and deposition on anode, finally triggering high surface area lithium (HSAL) formation due to locally enhanced resistance. Here, LiPF_6_, LiBF_4_, lithium difluoro(oxalate)borate (LiDFOB), lithium bis(oxalate)borate (LiBOB), lithium bis(fluorosulfonyl)imide (LiFSI), and lithium bis(trifluoromethanesulfonyl)imide (LiTFSI) in carbonate‐based solvents are investigated in LiNi_0.6_Co_0.2_Mn_0.2_O_2_ (NCM 622) || graphite pouch cells with 4.5 V UCV. Despite the lower oxidative stabilities of LiBF_4_ and LiDFOB, thus enhanced HF formation, TM dissolution, and consequently electrode cross‐talk, higher capacity retention is observed compared to the case of LiPF_6_ electrolyte. Counterintuitively, it is not the TM deposit amount but rather the Li plating morphology that governs capacity fade, as these salts cause more uniform and compact lithium plating, i.e., lower surface area. In contrast, the dendritic HSAL with LiPF_6_ has a higher surface area, and more parasitic reactions, thus active Li (“Li inventory”) losses and capacity fade. Although NCM initiates the failure cascade, the capacity losses and cycle life of high‐voltage LIBs are predominantly determined by the anode, in particular the Li plating morphology and the corresponding surface area.

## Graphical abstract

High voltage (>4.5 V) LiNi*_x_* Co*_y_* Mn_z_O_2_ (NCM)||graphite cells show rollover failure in the course of electrode crosstalk, i.e., transition metal (TM) dissolution from NCM and deposition on graphite. Interestingly, oxidatively less stable Li salts enhance the cycle life despite higher electrode crosstalk, as the morphology of plated Li has a lower surface area, thus less active lithium (capacity) losses.

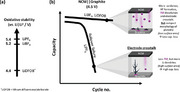

## 1 Introduction

Layered oxide‐based cathodes (LiNi*_x_* Co*_y_* Mn*_z_* O2; *x*+*y*+*z* = 1; NCM) with high Ni‐content, *e.g*., LiNi_0.6_Co_0.2_Mn_0.2_O_2_ (NCM 622), can enhance the specific energy of lithium ion batteries (LIBs) due to their higher specific capacity (>180 mAh g^−1^) and mean discharge potential (>3.7 V *vs*. Li|Li^+^).^[^ ^1^, ^2^ ^]^ Further enhancement can be achieved by increasing the upper cut‐off voltage (UCV, >4.3 V) which further increases both, the specific discharge capacity and mean voltage.^[^ ^3^, ^4^, ^5^ ^]^ However, high‐voltage operation of LIBs with the state‐of‐the‐art (SOTA) organic carbonate‐based electrolytes is challenging due to NCM structural degradation and transition metal (TM *=* Ni, Co, Mn) dissolution, leading to capacity fade.^[^ ^3^, ^5^, ^6^, ^7^, ^8^, ^9^ ^]^ The latter is particularly detrimental as dissolved TMs can deposit on the negative electrode over the course of electrode cross‐talk, leading to local resistance increase.^[^ ^3^, ^8^, ^10^, ^11^ ^]^ This phenomenon increases the risk of inhomogeneous Li plating and high surface area lithium (HSAL) formation,^[^ ^3^, ^7^, ^11^ ^]^ which poses operational safety hazards and reduces Li inventory/cell discharge capacity due to reactions of HSAL with the electrolyte, consequently leading to a more rapid capacity fade and in the worst‐case scenario to a “rollover” failure.^[^ ^12^, ^13^, ^14^ ^]^

Various approaches have been proposed to prevent electrode cross‐talk, e.g., via scavenging dissolved TMs in the electrolyte or via decreasing the amount of TM dissolution from the cathode in the first place. The former can be achieved by employing electrolyte additives such as lithium difluorophosphate (LiDFP; LiPO_2_F_2_),^[^ ^15^, ^16^, ^17^, ^18^, ^19^ ^]^ while the latter is enabled by electrolyte engineering or cathode modification such as employing electrochemomechanically more stable single crystals and/or particle coatings.^[^ ^20^, ^21^, ^22^ ^]^ With regards to electrolyte engineering, fluorinated electrolyte solvents are speculated to decrease TM dissolution by decreasing electrolyte oxidation and the intertwined HF formation.^[^ ^23^, ^24^, ^25^, ^26^ ^]^ Replacing the conventional LiPF_6_ conducting salt with LiBF_4_ is also shown to suppress TM dissolution from delithiated NCM 111 during storage experiments.^[^ ^27^ ^]^ Furthermore, employing LiBF_4_ is demonstrated to improve capacity retention in NCM 622 || graphite cells operated up to 4.35 V,^[^ ^28^ ^]^ despite the lower anodic stability of LiBF_4_.^[^ ^29^, ^30^ ^]^

The contrasting correlation between anodic stability and cycle life highlights the possibility of a more dominant factor than electrode cross‐talk that governs the overall cycling stability of high‐voltage LIBs when an alternative lithium salt is employed. Given that, this work aims to elucidate this factor by investigating common lithium salts, i.e., LiPF_6_, LiBF_4_, LiB(C_2_O_4_)_2_ (lithium bis(oxalato)borate; LiBOB), LiBF_2_(C_2_O_4_) (lithium difluoro(oxalato)borate; LiDFOB), lithium bis(trifluoromethane)sulfonimide (LiTFSI), and lithium bis(fluorosulfonyl)imide (LiFSI), dissolved in a conventional solvent mixture of ethylene carbonate (EC) and ethyl methyl carbonate (EMC), for NCM 622 || graphite cells charged to an UCV of 4.5 V. In particular, the impact of different lithium salts on capacity retention, TM dissolution, and lithium metal plating behavior/morphology is investigated to get further insights into the complex nature of electrode cross‐talk and rollover failure processes in high‐voltage/energy LIBs.

## 2 Results and Discussion

### 2.1 Electrochemical Investigations

**Figure 1:**
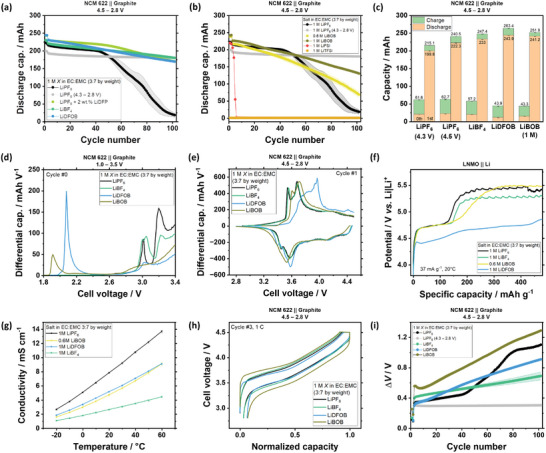
a) Discharge capacity versus cycle number of cells with 1 m LiPF_6_ ‐based electrolytes charged to UCVs of 4.3 and 4.5 V and compared with 1 m LiBF_4_, 1 m LiDFOB, and 1 m LiPF_6_ + 2 wt% LiDFP as well as b) 0.6 m LiBOB, 1 m LiBOB, 1 m LiFSI, and 1 m LiTFSI. c) Charge and discharge capacities in the 0th and 1st cycle. d) 0th and e) 1st cycle differential capacity versus voltage plot. f) Overcharge experiments for various electrolytes in LNMO || Li cells to establish anodic stability limit. e) Ionic conductivity of 1 m LiPF_6_, 0.6 m LiBOB, 1 m LiDFOB, and 1 m LiBF_4_ at a temperature range of −20–60 °C. f) Normalized voltage profile, g) polarization growth of cells with LiPF_6_, LiBOB (1 m), LiDFOB, and LiBF_4_

**Figure** **1**a depicts the discharge capacity of NCM 622 || graphite pouch cells with electrolytes containing LiPF_6_, LiBF_4_, and lithium difluoro(oxalate)borate (LiDFOB) as conducting salts in an EC‐EMC solvent mixture (3:7 by wt), galvanostatically charged to an upper cut‐off voltage (UCV) of 4.5 V. Cells with electrolytes containing lithium bis(oxalate)borate (LiBOB), lithium bis(fluorosulfonyl)imide (LiFSI), and lithium bis(trifluoromethane)sulfonylimide (LiTFSI), exhibit poorer capacity retention than cells with electrolytes containing LiBF_4_ and LiDFOB as shown in Figure 1b. Due to the limited solubility of LiBOB, an electrolyte mixture with 0.6 M rather than 1.0 M LiBOB is considered for comparison (Figure S1, Supporting Information). Cells cycled with LiTFSI and LiFSI electrolytes are shown for comparison but not considered for further analysis due to their inability to deliver reasonable capacities, which can be attributed to aluminum current collector dissolution (Figure S2, Supporting Information).^[^ ^31^ ^]^

Figure 1a also displays enhanced capacity retention enabled by adding 2 wt% lithium difluorophosphate (LiDFP; optimization in Figure S3, Supporting Information), which is considered an effective electrolyte additive for high‐voltage LIBs.^[^ ^14^, ^17^, ^32^ ^]^ However, despite the prevention of rollover failure, accelerated capacity fading is still observed in cells with LiDFP after ≈40 cycles, as these cells still suffer from Li metal plating, despite the suppressed TM deposition via TM scavenging in the presence of LiDFP (Figure S4, Supporting Information). The higher capacity retention and less Li metal plating for these cells at a lower C‐rate of 0.5 C (Figures S3 and S4, Supporting Information) indicate kinetic limitations of the negative electrode in NCM 622 || graphite cells.

In this work, the formation cycles consist of one half‐charge (pre‐cycle, 0^th^ cycle, 3.5 V UCV) and two full‐charge cycles (1^st^ and 2^nd^ cycle; 4.5 V UCV, Figure S5a, Supporting Information), based on previous reports.^[^ ^33^, ^34^ ^]^ In Figure 1a–c, cells cycled with LiBF_4_, LiDFOB, and LiBOB deliver higher 1^st^ cycle discharge capacities compared to LiPF_6_, which partially can stem from a lower capacity loss in the 0^th^ cycle. As shown in Figure 1c, the 0^th^ cycle charge capacities of cells with LiBF_4_, LiDFOB, and LiBOB salt are lower than LiPF_6_, suggesting less capacity consumption for solid electrolyte interphase (SEI) formation, which subsequently leads to lower capacity loss (Figure S6a, Supporting Information). In more detail, the differential capacity versus voltage plots in Figure 1d show distinct reduction peaks for cells with different conducting salts. In cells with LiPF_6_, the peaks at 3.0 and 3.2 V originate from the reduction of electrolyte solvents,^[^ ^30^, ^35^, ^36^, ^37^ ^]^ which is also observed in cells with LiBF_4_. In contrast, LiDFOB and LiBOB salts reduce the peak intensities from solvent reduction, likely because of earlier reduction of the DFOB^‐^ and the BOB^‐^ anion, at 2.1 and 1.9 V, respectively.^[^ ^38^ ^]^

As shown in Figure 1c, *NCM 622 || graphite* cells with LiBF_4_ and LiDFOB have higher 1st cycle charge capacities, hinting at electrolyte oxidation, resulting in additional, electrolyte oxidation‐derived, active Li, which would subsequently intercalate into the graphite negative electrode (Figure S6, Supporting Information).^[^ ^39^ ^]^ This can be supported by the additional peak at ≈4.0 V in the differential capacity versus voltage plot (Figure 1e, voltage profile in Figure S7b, Supporting Information) and the lower initial potential plateau at ≈4.4 V seen in the overcharge experiment (Figure 1f).^[^ ^40^, ^41^ ^]^ Three‐electrode investigation further reveals that the larger capacities in cells with LiDFOB occur without an upshift in NCM potential during charging (Figure S8a, Supporting Information). Interestingly, when the LiDFOB electrolyte is employed for *NCM 622 || Li* cells, no additional capacity is observed (Figure S8a,b, Supporting Information). Possibly, lithium oxalate produced from LiDFOB reduction on graphite can diffuse toward the positive electrode and get oxidized,^[^ ^42^, ^43^ ^]^ whereas the oxalate group formed on Li metal tends to be bonded stronger to the SEI.^[^ ^44^ ^]^ However, a detailed elucidation of the difference in charge capacity between the types of cell chemistry remains open.

In the subsequent 1 C charge/discharge cycles, the discharge capacities delivered by cells with electrolytes containing LiBF_4_, LiDFOB, and LiBOB are higher than those with LiPF_6_ (Figure 1a,b), despite the lower ionic conductivity of these alternative salts in EC‐EMC mixtures, shown in Figure 1g. This implies that an ionic conductivity as low as ≈2.7 mS cm^−2^ (1 m LiBF_4_ in at 20 °C) is sufficient for cell operation at a practical current density of ≈2 mA cm^−2^.^[^ ^45^ ^]^ Nevertheless, each electrolyte affects the cell polarization behavior differently. In the charging voltage profile depicted in Figure 1h, LiBOB results in the most pronounced increase of overvoltage and voltage hysteresis, followed by LiBF_4_. In contrast, LiDFOB decreases the overvoltage compared to LiPF_6_, suggesting better Li‐ion conducting interphases, i.e., SEI on the anode, as discussed.^[^ ^38^, ^46^ ^]^

Upon further galvanostatic cycling, rapid capacity fading, referred to as the “rollover” failure, is observed in cells with LiPF_6_ (Figure 1a,b), which is typical for NCM || graphite cells operated to an UCV of 4.5 V.^[^ ^3^, ^47^ ^]^ Surprisingly, rollover failure is not observed in cells with electrochemically less stable electrolytes, i.e., LiBF_4_ and LiDFOB. The rollover failure correlates with severe polarization growth, though the polarization is still lower than in cells with LiBOB as shown in Figure 1i.

### 2.2 Capacity Endpoint Slippage Analysis

**Figure 2:**
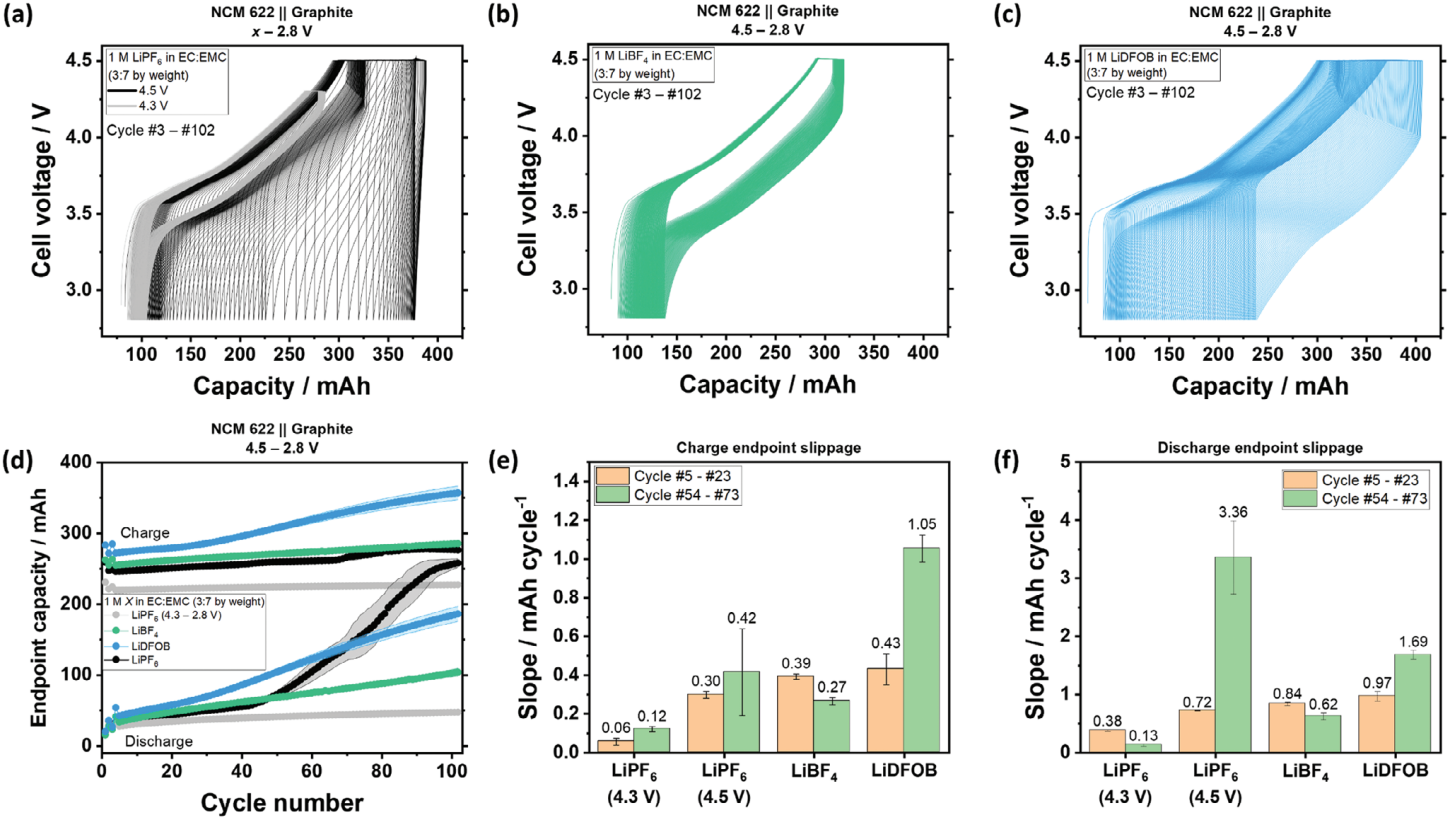
Evolution of voltage profiles of cells with a) LiPF_6_, b) LiBF_4_, and c) LiDFOB, from cycle 3 to 102. g) Endpoint capacity versus cycle number plot, h) charge endpoint slippage slope, and f) discharge endpoint slippage slope of cells with LiPF_6_, LiDFOB, and LiBF_4_.

The voltage profile slippage of cells with LiPF_6_ and the best‐performing electrolytes, i.e., LiBF_4_ and LiDFOB, are plotted in **Figure** **2**a–c. Here, charge and discharge endpoint slippage refers to the continuous shift of charge and discharge endpoint to higher capacities, respectively.^[^ ^39^, ^48^ ^]^ The former may stem from side reactions on the positive electrode, e.g., electrolyte oxidation, short circuits, or shuttling mechanisms.^[^ ^39^, ^48^, ^49^ ^]^ The latter shifts according to the charge slippage and additionally may shift in the course of parasitic reactions on the negative electrode that consumes active Li, e.g., reduction reactions.^[^ ^39^, ^48^ ^]^ The slippages can be regarded as an indicator for traces of parasitic reactions, which would not be observed in a single charge/discharge cycle.

The high capacity retention of cells with LiPF_6_ charged to an UCV of 4.3 V correlates with voltage profile overlaps and low slippage from cycle 3 to 102 (Figure 2a).^[^ ^39^, ^48^ ^]^ In contrast, for an UCV of 4.5 V, a larger charge and discharge endpoint slippage is observed, with the latter showing visibly large leaps/gaps in Figure 2a,^[^ ^39^, ^48^ ^]^ which can be correlated with rollover failure in Figure 1a. The large increase in discharge endpoint slope during the rollover failure (cycle #54‐#73, Figure 2f) stems from severe active lithium loss due to electrolyte decomposition triggered by HSAL and TM deposits,^[^ ^3^, ^10^, ^39^, ^48^ ^]^ as well as dead lithium formation.^[^ ^50^, ^51^ ^]^ Although both, SEI formation on HSAL and dead lithium contribute to lithium loss, the former is argued to be more severe.^[^ ^51^ ^]^

Cells with electrolytes containing LiBF_4_ and LiDFOB show similar charge endpoint slippage in the first ≈20 cycles, both higher than LiPF_6_ (Figure 2e), pointing to more parasitic reactions.^[^ ^48^, ^52^ ^]^ Cells with the LiDFOB electrolyte have the highest increase in charge capacity slippage after 40 cycles, which may be related to particle cracking (Figure S9, Supporting Information) and the higher tendency of LiDFOB to oxidize (Figure 1f)^[^ ^48^, ^52^, ^53^ ^]^ Accordingly, a large amount of gas, likely to be CO_2_,^[^ ^54^, ^55^ ^]^ is generated (Figures S10 and S11, Supporting Information), and the DFOB^‐^ concentration gets depleted (Figure 4a). The low discharge slippage of cells with LiBF_4_ aligns with their high capacity retention and can be related to low active lithium loss in the negative electrode (Figure 2f). For cells with LiDFOB, large discharge and charge endpoint slippages, with good capacity retention, are observed. This can be interpreted as a continuous replacement of lithium losses in the negative electrode by active lithium generation from electrolyte oxidation.^[^ ^39^, ^40^ ^]^

### 2.3 Post Mortem Cathode Electrochemistry

**Figure 3:**
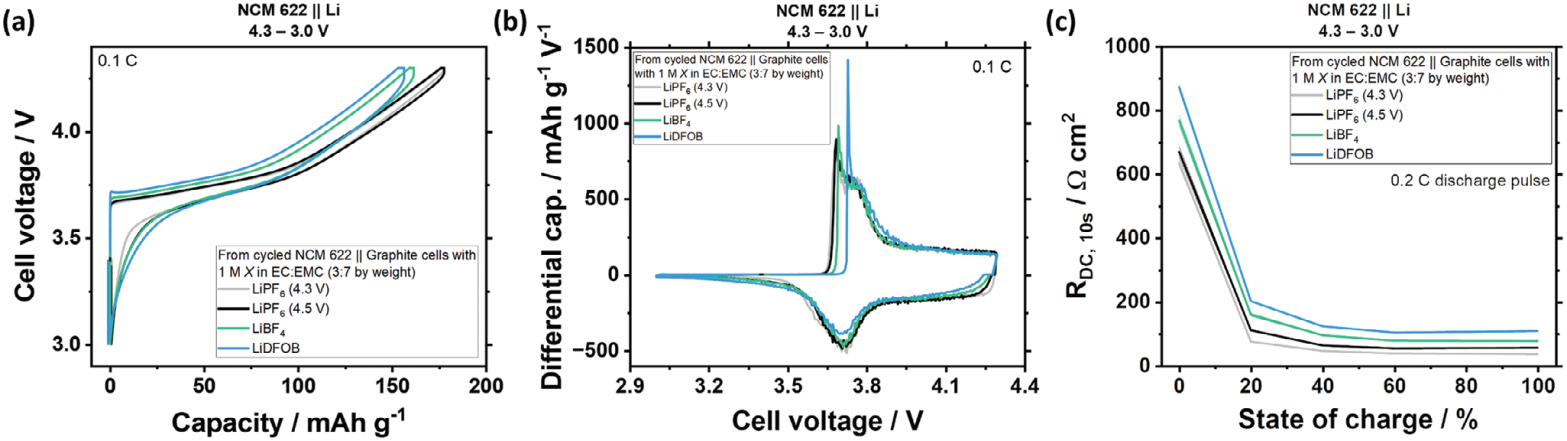
Cycled NCMs from graphite‐based cells reassembled in fresh lithium cells. a) Cell voltage versus capacity, b) differential capacity versus voltage, and c) DCIR versus SoC plots of NCM 622 || Li cells with NCM 622 extracted from NCM 622 || graphite cells cycled with LiPF_6_, LiBF_4_, and LiDFOB‐based electrolytes.

NCM 622 electrodes are disassembled from NCM 622 || graphite cells after 102 cycles and reassembled as NCM 622 || Li cells to investigate NCM degradation. **Figure** **3**a shows the highest capacities and no relevant changes in voltage profiles for NCM from cells with LiPF_6_ cycled to UCVs of 4.5 and 4.3 V, suggesting still stable NCM 622, which is in line with differential capacity plots in Figure 3b and resistances from 0 to 100% state of charge (SoC) range in Figure 3c. The resistance values are highest for electrodes from cells with LiDFOB, followed by LiBF_4_, and LiPF_6_, correlating with the oxidative stability of the electrolytes in Figure 1f, suggesting surface degradation induced by electrolyte oxidation.^[^ ^56^ ^]^ Cathode damage can consequently be excluded to cause the huge capacity fade in *graphite*‐based full cells in Figure 1f.

Although NCM 622 from cells with the LiDFOB electrolytes has a thicker cathode electrolyte interphase (CEI) layer compared to the case of LiPF_6_ and LiBF_4_ electrolytes (Figure S12d, Supporting Information), due to more electrolyte oxidation, (Figure 2e) this contrasts previous reports showing the benefit of employing LiDFOB as an electrolyte additive to form protective CEI from LiDFOB oxidation.^[^ ^46^, ^57^ ^]^ When LiDFOB is used as an electrolyte additive, it is fully consumed in the early cycles and the remaining LiPF_6_ salt remains as a single Li salt, which in turn is anodically more stable and does not oxidize. In contrast, using LiDFOB as the *main conducting salt* is detrimental toward the cathode as it is still present in the electrolyte even after CEI formation and continuously oxidizes.^[^ ^54^, ^58^, ^59^ ^]^

### 2.4 Electrolyte Decomposition

**Figure 4:**
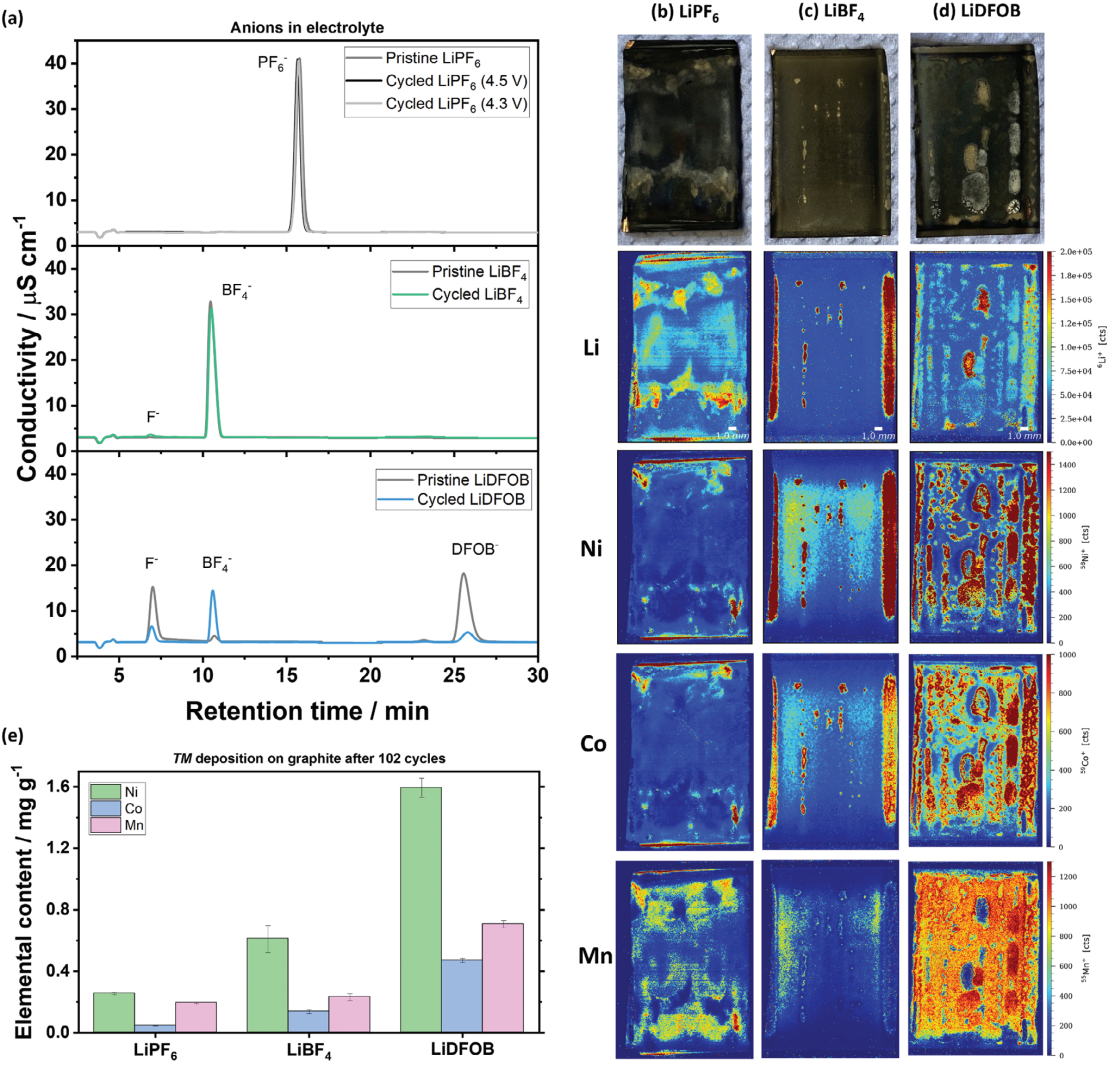
a) Anion‐separated chromatography for electrolytes with LiPF_6_, LiBF_4_, and LiDFOB as conducting salts. The initially high amount of F^‐^ in the pristine LiDFOB electrolyte likely stems from the thermodynamically favorable LiDFOB hydrolysis.^[^ ^61^ ^]^ LA‐ICP‐MS element mapping results (Li, Ni, Co, Mn) for cells with b) LiPF_6_, c) LiBF_4_, d) LiDFOB of selected negative electrodes after 102 charge/discharge cycles. e) Quantification of TM content on the negative electrodes after 102 cycles obtained via ICP‐OES.

To elucidate the parasitic reactions, the electrolytes are extracted from the cells after 102 charge/discharge cycles and investigated via ion chromatography with conductivity detection. The large decrease in DFOB^‐^ peak intensity in **Figure** **4**a indicates pronounced decomposition, as speculated from the charge endpoint slippage data above (Figure 2f–h). Although the reduction of DFOB^‐^ during SEI formation on graphite (Figure 1d) and Li metal also plays a role in lowering the DFOB^‐^ peak intensity,^[^ ^43^ ^]^ the rise of BF_4_^‐^ peak intensity suggests that DFOB^‐^ is continuously oxidized on the positive electrode according to proposed reaction mechanisms (Figure S10, Supporting Information).^[^ ^46^, ^55^, ^58^, ^59^, ^60^ ^]^ This is also supported by a large amount of gas, likely to be CO_2_, produced after 102 charge/discharge cycles (Figure S11, Supporting Information).^[^ ^55^, ^58^ ^]^ In contrast, the LiBF_4_ electrolyte with higher anodic stability than LiDFOB (Figure 1f) is only slightly decomposed, as indicated by the presence of F^‐^ from BF_4_^‐^ hydrolysis (Figure 4a).^[^ ^61^ ^]^ The lack of F^‐^ in the LiPF_6_ electrolyte cycled to an UCV of 4.5 V further suggests its slightly higher anodic stability than the LiBF_4_ electrolyte (Figure 1f).

### 2.5 Amount and Distribution of Transition Metal Deposits

Rectangular sections of the negative electrode rolls are taken after 102 charge/discharge cycles in a dry room atmosphere (Figure S13, Supporting Information) and investigated with laser ablation‐inductively coupled plasma‐mass spectroscopy (LA‐ICP‐MS; Figure 4b–d). Stark differences in the Li distribution can be seen, which tend to correlate with the distribution of Ni and Co deposits, particularly for cells with LiBF_4_ and LiDFOB.^[^ ^3^, ^7^ ^]^ Interestingly, while TM deposition in cells with LiPF_6_ and LiDFOB salts is distributed throughout the electrode, TM deposits accumulate more at the edge of the electrodes for cells cycled with LiBF_4_ salt, possibly due to the higher mechanical pressure in this region.^[^ ^62^ ^]^ According to a previous study,^[^ ^63^ ^]^ enhanced mechanical pressure results in a decrease in spatial distance between the positive and negative electrodes, and TM deposition in these areas is more likely due to shortened diffusion pathways. This is particularly more pronounced in cells with LiBF_4_, likely due to the stronger tendency of BF_4_^‐^ to associate with cations compared to PF_6_^‐^,^[^ ^64^ ^]^ leading to low ionic conductivity of LiBF_4_ (Figure 1g),^[^ ^64^, ^65^ ^]^ which makes it more sensitive toward diffusion distances.^[^ ^66^ ^]^ Without applied pressure, the pressure distribution in the jelly roll is altered and Li plating on graphite with LiBF_4_ no longer concentrates at the edges (Figure S14, Supporting Information).

Quantitative investigations via inductively coupled plasma‐optical emission spectroscopy (ICP‐OES, Figure 4e) reveal the highest amounts of TM deposits on graphite cycled with LiDFOB, followed by LiBF_4_ and LiPF_6_. This trend correlates with the charge and discharge endpoint slippage slope in the early cycles (Figure 2e,f). Likely, the slightly higher electrolyte oxidation (higher *charge* slippage) and the corresponding HF formation trigger TM dissolution.^[^ ^39^, ^67^ ^]^ As a result, larger amounts of TM deposits on the negative electrode trigger electrolyte decomposition and HSAL formation, increasing also the *discharge* endpoint slippage.^[^ ^3^, ^8^, ^39^ ^]^ Notably, salts with the highest capacity retention also show the highest amount of TM deposits on their respective negative electrodes.^[^ ^17^, ^18^ ^]^

### 2.6 Lithium Deposition Morphology

**Figure 5:**
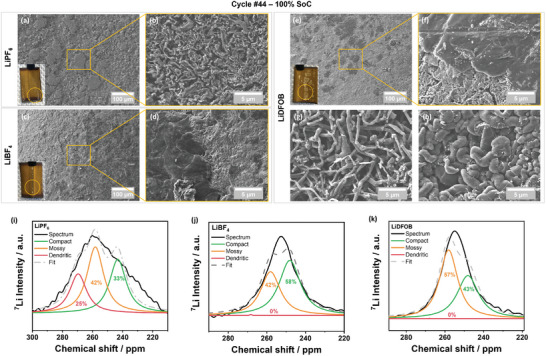
(a,c,e) Low and (b,d,f‐h) high magnification SEM images of fully charged negative electrodes obtained from cells with electrolytes containing LiPF_6_, LiBF_4_, and LiDFOB at cycle #44. The yellow circles in the insets of (a,c,e) indicate the region where the respective SEM images are taken from. (g) and (h) are taken from areas not shown in (e). NMR spectra of metallic Li on fully charged graphite negative electrodes from cells with (i) LiPF_6_, (j) LiBF_4_, and (k) LiDFOB, fitted with three peaks arising from different Li morphology.

Improved capacity retention despite higher amounts of TM deposits points to other factors relevant to cycle life and rollover failure. **Figure** **5** shows SEM images of negative electrodes harvested at cycle #44 and 100% state of charge (SoC; Figure S15, Supporting Information), directly after the rollover failure onset for cells with LiPF_6_ (Figure 1a). Figure 5a reveals that metallic lithium deposits, indicated by the circle in the inset, are only identified locally in relatively small amounts on graphite from a cell with LiPF_6_. These are characterized by high surface area, often referred to as dendritic lithium species (Figure 5b), which is in agreement with previous reports.^[^ ^3^, ^7^ ^]^ In contrast, graphite from cells with LiBF_4_ (Figure 5d) as well as LiDFOB (Figure 5f) exhibit lithium metal deposits with more compact morphologies, i.e., lower surface area. Compared to graphite from cells with LiBF_4_, more diverse metallic lithium morphologies are observed with LiDFOB (Figure 5f–h), similar to results obtained in “zero‐excess” Li metal batteries with LiDFOB‐based electrolytes.^[^ ^58^, ^59^ ^]^ On a broader scale, the presence of high surface area lithium (HSAL) is also evidenced by the presence of dark deposits on the separator retrieved from cells operated with LiPF_6_ and LiDFOB, due to HSAL sticking to the separators.^[^ ^7^ ^]^ These morphological differences in metallic lithium deposits seen at cycle #44 and 100% SoC are also observed at cycle #102 and 0% SoC (Figure S16, Supporting Information), indicating similarities between the reversible and irreversible fractions of deposited metallic lithium. Hence, the morphology of metallic lithium deposits rather depends on anion chemistry than the amount of dissolved TMs.

To establish the differences in metallic lithium morphology at a broader scale, parts of fully charged negative electrodes from cells at cycle #44 containing metallic lithium deposits are analyzed via ^7^Li nuclear magnetic resonance (NMR) spectroscopy. The obtained signals in the chemical shift region for metallic lithium species (245‐270 ppm) are deconvoluted using three peaks with restricted chemical shifts, representative of compact, mossy, and dendritic lithium metal deposits.^[^ ^68^ ^]^ Further discussion regarding the peak fitting quality can be found in Supplementary Note S1.

A comparison of Li salts in Figure 5i–k confirms the presence of dendritic lithium species only on graphite cycled with LiPF_6_. Furthermore, graphite cycled with LiBF_4_ has a higher proportion of compact lithium metal deposits compared to that cycled with LiDFOB, which is in line with the SEM images. Given that the LiBF_4_ salt does not participate in SEI formation as much as the LiDFOB salt (Figure 1d; Figure S12, Supporting Information),^[^ ^43^ ^]^ the dense lithium plating morphology likely stems from localized lithium plating in the high‐pressure regions at the edge regions of the jelly rolls (Figure 4c).^[^ ^58^, ^68^ ^]^

In cells cycled with LiDFOB, an increased proportion of mossy lithium metal deposits, along with higher amounts of TM deposits are observed, which is likely the origin of the larger discharge endpoint slippage compared to cells with LiBF_4_. However, in cells with LiDFOB, the participation of LiDFOB in SEI formation plays an important role in forming more compact lithium deposits compared to LiPF_6_ (Figure 1d; Figure S12, Supporting Information).^[^ ^43^, ^44^, ^59^ ^]^ It is argued that LiDFOB reduction produces nanosized LiF due to capping by oxalate species, which is important for compact lithium plating morphology.^[^ ^44^ ^]^ Finally, considering that rollover failure is only observed in cells operated with LiPF_6_, HSAL deposits with dendritic microstructures can be concluded to strongly cause accelerated lithium inventory losses leading to rollover failure, while more uniform and compact metallic lithium seems to be less detrimental.^[^ ^69^ ^]^ As such, the lithium plating morphology can be concluded to be strongly influenced by anionic species within the electrolytes.

## 3 Conclusion

In this work, charge/discharge cycling with varied lithium conducting salts in an ethylene carbonate (EC)/ethyl methyl carbonate (EMC) solvent mixture is investigated in *high voltage* (4.5 V) NCM 622 || graphite wounded pouch cells. Galvanostatic overcharge experiments, capacity endpoint slippage, laser ablation‐inductively coupled plasma‐mass spectrometry (LA‐ICP‐MS), and ICP‐optical emission spectroscopy (ICP‐OES) show decreased anodic stabilities, more electrode crosstalk (=TM dissolution from cathode and deposition on anode) and more lithium plating for LiBF_4_ and lithium difluoro(oxalate)borate (LiDFOB)‐based electrolytes compared to the conventional LiPF_6_ electrolyte, but, counterintuitively suppressed rollover failure and improved capacity retention.

**Figure 6:**
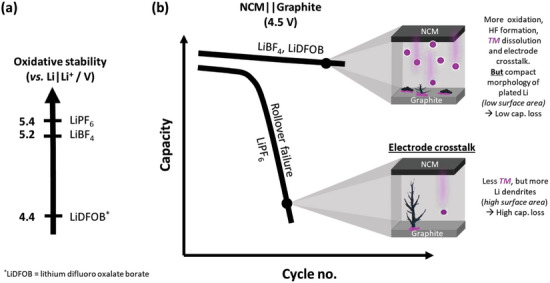
The lower oxidative stability of LiBF_4_ and LiDFOB compared to LiPF_6_‐based electrolytes in EC‐EMC solvent mixture (3:7 by wt), leads to higher HF formation, TM dissolution, and electrode cross‐talk. b) Despite higher electrode crosstalk, the cycle life and capacities are counterintuitively improved for high voltage NCM || graphite cells (4.5 V UCV) compared to LiPF_6_. A more uniform and compact Li plating morphology (*low* surface area) for cells with LiBF_4_ and LiDFOB, compared to dendritic Li plating (*high* surface area) for cells with the LiPF_6_ electrolyte, results in less parasitic reactions (e.g., with the electrolyte), less active Li loss, and consequently less capacity fading. The morphology and surface area of plated Li is obviously more crucial for high‐voltage applications than the amount of TM deposits. Partially redrawn from a previous report.^[^ ^18^ ^]^

A combined scanning electron microscopy (SEM) and nuclear magnetic resonance (^7^Li NMR) investigation of metallic lithium deposits on the negative electrode indicates different morphologies of plated lithium. While LiBF_4_ and LiDFOB exclusively exhibit uniform and compact (*low* surface area) lithium plating deposits, a dendritic lithium plating morphology (*high* surface area) is observed in cells cycled with LiPF_6_. The data presented in this work strongly points to the higher relevance of lithium plating morphology, thus surface area, on capacity losses than the amount of TM deposits (**Figure** **6**).

## 4 Experimental Section

### Electrolyte Formulation

Electrolytes were formulated by mixing 1 M of conducting salts, i.e., LiPF_6_ (battery grade; E‐Lyte Innovations GmBH), LiBF_4_ (battery grade; E‐Lyte Innovations GmBH), LiB(C_2_O_4_)_2_ (lithium bis(oxalato)borate; LiBOB; Sigma–Aldrich), LiBF_2_(C_2_O_4_) (lithium difluoro(oxalato)borate; LiDFOB; 99%; Abcr GmBH), lithium bis(trifluoromethane)sulfonimide (LiTFSI; 99.5%; Solvonic), and lithium bis(fluorosulfonyl)imide (LiFSI; 99.5%; Solvonic), in an ethylene carbonate (EC) and ethyl methyl carbonate (EMC) solution mixture (3:7 by weight; battery grade; E‐Lyte Innovations GmBH). Electrolytes containing LiPO_2_F_2_ (lithium difluorophosphate; LiDFP; Chemfish Tokyo Co., Ltd.) were prepared by adding 0.5, 1, 2, or 4 wt% LiDFP to the LiPF_6_‐based electrolyte. All electrolytes were prepared in an argon‐filled glovebox (MBraun) with H_2_O and O_2_ levels below 1 ppm.

### Cell Setup

Wounded multi‐layer NCM 622 || graphite pouch cells (LiFun Technologies) balanced for an UCV of 4.5 V, with a nominal capacity of 200 mAh, were used for electrochemical investigations. The positive electrode has a total mass loading of 12.2 mg cm^−2^, consisting of 96.4 wt% NCM 622 polycrystalline powder. The negative electrode has a total mass loading of 9.2 mg cm^−2^, consisting of 95.7 wt% artificial graphite_._ Prior to electrolyte filling, the cells were dried at 80 C under reduced pressure (<0.05 mbar) for 12 h. Afterward, the cells were filled with 0.75 mL of electrolytes and sealed by a heat‐crimper at 165 C for 5 s at a relative pressure of ‐80 kPa using a vacuum sealer (GN‐HS200 V, Gelon Lib Group). For electrochemical investigation, custom cell holders capable of applying 2 bar of pressure were used.^[^ ^34^ ^]^

Overcharge experiments were carried out in three‐electrode setups in T‐cells (Swagelok). LiNi_0.5_Mn_1.5_O_2_ (>99%; Sigma–Aldrich)‐based composite electrode (Ø12 mm) with an areal capacity of ≈0.36 mAh cm^−2^, made up of 90 wt% active material, 5 wt% carbon black (Super C65, Imerys Graphite & Carbon), and 5 wt% poly(vinylidene difluoride) binder (Solef 5130, Solvay) coated on aluminum foil, was used as the working electrode. For the counter and reference electrodes, Ø12 mm and Ø8 mm lithium foil (500 µm; Gelon Lib Group) were used, respectively. The working and counter electrodes were separated by three layers of Ø12 mm polypropylene (PP) fiber separators (FS2190; Freudenberg) soaked in 120 µL of electrolyte and the reference electrode is separated by two layers of the same separator soaked in 120 µL of electrolyte. Pouch cells were sealed and T‐cells were assembled in a dry room atmosphere with a dew point of at least −50 °C (0.16% relative humidity).

NCM 622 || Li cells were investigated in a two‐electrode configuration in two‐electrode coin cells (CR2032, Hohsen Corporation). The positive electrodes (Ø12 mm) were taken from the positive electrode roll of the wounded pouch cells and the negative electrodes were Ø15 mm Li metal (500 µm thick, Gelon). The electrodes were separated with one layer of Celgard 2500 separator (Ø16 mm) soaked with 50 µL of electrolyte.

Three‐electrode NCM 622 || graphite cells were investigated in T‐cells (Swagelok). The positive (Ø12 mm) and negative electrodes (Ø12.5 mm) were taken from the positive and negative electrode rolls of the wounded pouch cells, respectively. The electrodes were separated with one layer of Celgard 2500 separator (Ø13 mm) soaked with 50 µL of electrolyte and lithium metal was used as the reference electrode. At least two coins, pouches, and T‐cells were assembled to assess reproducibility.

*Post‐mortem* electrochemical investigations of NCM 622 were carried out in two‐electrode coin cells. Cycled NCM 622 electrode rolls were extracted from NCM 622 || graphite cells after 102 charge/discharge cycles. The rolls were punched into circular discs (Ø12 mm) and then assembled as NCM 622|| Li cells. One layer of Celgard 2500 separator soaked with the LiPF_6_ electrolyte was used.

### Electrochemical Investigation

The electrochemical performance of NCM 622 || graphite pouch cells was evaluated using a Maccor 4000 battery testing system. After sealing, the cells were charged to 1 V and held at this voltage for 20 h to ensure adequate wetting. For solid electrolyte interphase (SEI) formation, the cells were charged to 3.5 V at a constant current (CC) of 10 mA (0.05 C), held at a constant voltage (CV) of 3.5 V for 1 h, and then discharged to 2.8 V with a CC of 10 mA (0.05 C). This cycle is considered as cycle zero. The cells then underwent two charge/discharge cycles with upper cut‐off voltages (UCVs) of 4.3 or 4.5 V. For these two cycles, the cells underwent CC charging step at 40 mA (0.2 C), followed by a CV step until the current drops to <4 mA (0.02 C), and finally CC discharge step at 40 mA (0.2 C) to 2.8 V. After the formation cycles at 20 C, the cells were subjected to additional 100 charge/discharge cycles at 20 C or high‐temperature storage experiment at 60 C. For the former, unless otherwise stated, the cells were subjected to CC charging step at 200 mA (1 C) to, followed by a CV step until the current dropped to <10 mA (0.05 C) or 300 mAh (1.5 C) of capacity is reached, and finally CC discharge step at 200 mA (1 C). At the last cycle, the cells were further discharged with a CV step at 2.8 V until the current dropped to <10 mA (0.05 C). For the storage experiment, the cells were first degassed after formation cycles, rested for 6 h at 60 C, charged with CC mode at 100 mA (0.5 C) to 4.5 V, followed by a CV step until the current drops to <10 mA (0.02 C). The cells were then rested for 720 h and their open‐circuit voltage (OCV) was measured. Finally, the cells were discharged with CC mode at 100 mA (0.5 C) to 2.8 V. At least two pouch cells per testing parameter were used to validate reproducibility.

Overcharge experiments were carried out after letting the T‐cells rest for 3 hr after cell assembly to allow for adequate electrolyte wetting. The cells were then charged with a CC mode (0.16 C, 1 C defined as 145 mA g^−1^) and the potential of the working electrode was measured. Charging is terminated when 30 h of measurement time or 6 V is reached. At least two T‐cells per electrolyte mixture were used to validate reproducibility.

The investigation of NCM 622 || Li cells utilized a similar cycling protocol as NCM 622 || graphite cells with two minor adjustments. First, the 0^th^ cycle pre‐charge step was eliminated. Second, the UCV and the LCV were adjusted to 4.6 and 2.9 V, respectively. The investigation of NCM 622 || graphite T‐cells utilized the same cycling protocol as NCM 622 || graphite pouch cells with two minor adjustments. In both cases, the 1 C value was set to 200 mA g^−1^. Two coins and T‐cells per electrolyte fomulation were assembled to validate reproducibility.

For *post mortem* electrochemical investigations, NCM 622 || Li cells were rested for 6 h after cell assembly. Then the cells underwent two charge/discharge cycles in a voltage window of 4.3–3.0 V. For these two cycles, the cells underwent CC charging step at 0.1 C (1 C is defined as 180 mA g^­1^) followed by a CV step until the current drops to <0.05 C (1 h cutoff), and finally CC discharge step at 0.1 C. For resistance measurements, the same cells underwent CC charging step at 0.05 C to 4.3 V, rested for 1 h, and then CC discharge pulse was applied for 10 s at 0.2 C. After 30 s of rest, the cells were then further discharged to 80% SoC with a 0.05 C CC discharge step and underwent the same resting and discharge pulse step. This procedure was repeated until 0% SoC.

### Electrolyte Conductivity Measurements

Electrolyte conductivity measurements were carried out under an inert atmosphere inside a glovebox (MBraun, H_2_O, and O_2_ <1 ppm). Conductivity cells were filled with the various electrolyte formulations as previously described.^[^ ^70^ ^]^ Cell constants were determined using a 0.01 m solution of KCl in H_2_O at 20 °C (VWR, known conductivity of 1.276 mS cm^−1^), averaged over five measurements. Disposable 2 mL Eppendorf Safe‐Lock Tubes were used as sample containers and each was filled with 750 µL of electrolyte. Impedance measurements were conducted on a Metrohm Autolab/M204 potentiostat/galvanostat with 12 channels and an 8‐channel multiplexer for a total of 96 channels in the frequency range of 50–20000 Hz using in‐house developed electrodes. The conductivity cells were placed in a temperature chamber (Memmert TTC256, 0.1 °C temperature setting accuracy) and each temperature was held for 2 h before measurement for equilibration. The ionic conductivity of the electrolytes was measured in the temperature range of −30–60 °C in 10 °C steps. Impedance spectra were fitted using a model specified with set parameters for resistors Rs and Rp, as well as for the constant phase element (CPE) with the Metrohm Nova software. Fitting was carried out after each additional measuring point by using the fitting model Rs(CPE − Rp). Electrolyte conductivity values were obtained from the quotient of the cell constant and the determined electrolyte resistance.

### Laser Ablation‐Inductively Coupled Plasma‐Mass Spectrometry (LA‐ICP‐MS)

LA‐ICP‐MS imaging measurements were performed using a 193 nm ArF excimer laser (Analyte Excite Excimer LA‐System, Teledyne Cetac, USA) coupled to an ICP‐MS system (7700 Series, Agilent Technologies, USA) via an Aerosol Rapid Introduction System (ARIS, Teledyne CETAC Technologies, USA). The laser was operated at a frequency of 50 Hz with a spot size of 50 µm, 12.5x scan speed (625 µm s^−1^), and laser fluence of 3 J cm^−2^.

### Inductively Coupled Plasma‐Optical Emission Spectroscopy (ICP‐OES)

Electrode samples were digested in reversed *aqua regia* using a microwave system (Multiwave 7000, Anton Paar, Graz, Austria) prior to analysis by inductively coupled plasma‐optical emission spectroscopy (ICP‐OES). The measurements were performed on an ARCOS (Spectro Analytical Instruments GmbH, Kleve, Germany) instrument, equipped with a scott‐style spray chamber, a cross‐flow nebulizer, and an axial positioned plasma torch. The following emission lines were observed during analysis: Li (670.780 nm), Ni (221.648, 231.604, and 232.003 nm), Co (228.616 nm, 237.862 nm, 238.892 nm), Mn (257.611 nm, 259.373 nm), Cu (224.700, 324.754, and 327.396 nm). The method and further parameters were adapted from Vortmann‐Westhoven et al. and Evertz et al.^[^ ^71^, ^72^ ^]^

### Ion Chromatography‐Conductivity Detection‐Mass Spectrometry (IC‐CD‐MS)

Analysis of anionic species was executed on an 850 Professional IC (Metrohm) with conductivity detection (CD). A Metrosep A Supp 7 column (250 × 4.0 mm, 5 µm; Metrohm) with a Metrosep A Supp 5 Guard/4.0 guard column was used for an isocratic separation of the analytes at an oven temperature of 65 °C and an applied flow rate of 0.7 mL min^−1^ over a total runtime of 30 min. The eluent consisted of a 3.6/3.4 mM Na_2_CO_3_/NaHCO_3_ aqueous solution and acetonitrile in a ratio of 58:42 (v/v). All samples were diluted 1:1000 with acetonitrile prior to investigation and the injection volume was set to 65 µL. The utilized suppressor was sequentially regenerated by 0.1 M sulfuric acid and rinsed with MilliQ Water in a 30 min interval. Instrument control, data acquisition, and data evaluation were performed with MagIC Net 3.3 (Metrohm).

### Scanning Electron Microscopy (SEM)

SEM (Carl Zeiss AURIGA; Carl Zeiss Microscopy GmbH) was employed to analyze the cross‐section and surface morphology of aged NCM 622 and graphite electrodes, respectively. EDX was performed at an accelerating voltage of 6 kV using an X‐MaxN 80mm^2^ EDX detector (Oxford Instruments). INCA software was used to assess the spectra (Oxford Instruments). Cells were disassembled in a dry room atmosphere with a dew point of at least ‐50 °C (0.16% relative humidity). For cross‐section SEM imaging, electrodes were cut using an ion beam cross‐section polisher (JEOL). Prior to the cross‐section cut and SEM analysis, 2 mL of dimethyl carbonate (DMC) was used to wash the electrode. To avoid moisture contact, samples were transferred using an air‐tight sample holder to the SEM analysis chamber.

### ^7^Li Nuclear Magnetic Resonance (NMR) Spectroscopy

Graphite negative electrodes were harvested from the pouch cells in an Argon‐filled Glovebox (MBraun, O_2,_ and H_2_O < 1 ppm) at the 42nd cycle at 100% SOC. Inside a dry room atmosphere with a dewpoint of at least −50 °C (0.16% relative humidity), a rectangular part from the respective electrode having observable amounts of metallic lithium deposited on top was cut to have the dimensions of 2 cm × 1 cm. The electrode was then vacuum‐sealed in a PE/PP cell housing in order to be transferable to the NMR probe without exposure to ambient conditions. Static ^7^Li NMR measurements were performed on a 4.7 T (200 MHz) Bruker Avance III spectrometer using a custom‐made probe suitable for the investigation of pouch cell samples. Prior to the NMR measurements of the actual samples, the ^7^Li NMR chemical shift scale was referenced to 0 ppm using a 1 mol L^−1^ LiCL + 1 g L^−1^ CuSO_4_ in water standard. For LiBF_4_ and LiDFOB samples, 1024 scans separated by a recycle delay of 0.5 s were averaged using pre‐saturation and an optimized π/2 pulse with a length of 6.0 µs (reflecting radio‐frequency field strengths of 41.6 kHz) and an amplitude of 80 W. For the LiPF_6_ sample, the number of scans was increased to 131 072 to achieve a sufficient signal‐to‐noise ratio while maintaining all other parameters. The FID was recorded with a dwell time of 0.1 µs and a time domain of 32 k. To obtain insights into the lithium morphology, ^7^Li NMR spectra were deconvoluted with a customized MatLab script based on peak fit.m by Thomas C. O'Haver.^[^ ^73^ ^]^ In order to verify the chemical shift for more uniform and compact metallic lithium deposits in the presence of charged graphite, an as‐manufactured lithium metal electrode (Honjo) with a thickness of 20 µm on 10 µm copper was placed on top of a charged graphite electrode, exhibiting a chemical shift of 244.5 ppm. Thus, ^7^Li NMR spectra obtained for the different samples were fitted using three Voigt functions that are restrained to have chemical shifts representing more uniform and compact (243–248 ppm), mossy (258–263 ppm), and dendritic (268–273 ppm) lithium deposit morphologies, as previously reported.^[^ ^68^ ^]^ The respective fractions for the morphology of metallic lithium were then calculated by relating the respective integral to the total ^7^Li NMR signal integral.

### X‐Ray Photoelectron Spectroscopy (XPS)

Investigations of the electrode‐electrolyte interphase were conducted using an Axis Ultra DLD XPS (Kratos Analytical) with a monochromatic Al Kα source (hν = 1486.6 eV) at an emission current of 10 mA with an accelerating voltage of 12 kV. Positive charging of the sample was suppressed by using a charge neutralizer. Cells were disassembled in the discharged state in a dry room atmosphere with a dew point of at least −50 °C (0.16% relative humidity) to avoid contamination from ambient air. Electrodes were analyzed after washing with dimethyl carbonate. Three spots per electrode were analyzed to evaluate reproducibility. Data were analyzed by CasaXPS (v2.3.23, Casa Software) and referenced to the hydrocarbon signal C 1s corresponding to 284.8 eV.

## Conflict of Interest

The authors declare no conflict of interest.

## Supporting information



Supporting Information

## Data Availability

The data that support the findings of this study are available from the corresponding author upon reasonable request.

## References

[1] R. Schmuch , R. Wagner , G. Hörpel , T. Placke , M. Winter , Nat. Energy 2018, 3, 267.

[2] H.‐J. Noh , S. Youn , C. S. Yoon , Y.‐K. Sun , J. Power Sources 2013, 233, 121.

[3] S. Klein , P. Bärmann , T. Beuse , K. Borzutzki , J. E. Frerichs , J. Kasnatscheew , M. Winter , T. Placke , ChemSusChem 2021, 14, 595. 33105061 10.1002/cssc.202002113 PMC7894331

[4] J. Kasnatscheew , R. Wagner , M. Winter , I. Cekic‐Laskovic , Top. Curr. Chem. 2018, 376, 16. 10.1007/s41061-018-0196-1 29671099

[5] J. Kasnatscheew , M. Evertz , B. Streipert , R. Wagner , S. Nowak , I. Cekic Laskovic , M. Winter , J. Power Sources 2017, 359, 458.

[6] J. Kasnatscheew , S. Röser , M. Börner , M. Winter , ACS Appl. Energy Mater. 2019, 2, 7733.

[7] S. Klein , P. Bärmann , L. Stolz , K. Borzutzki , J.‐P. Schmiegel , M. Börner , M. Winter , T. Placke , J. Kasnatscheew , ACS Appl. Mater. Interfaces 2021, 13, 57241. 34813694 10.1021/acsami.1c17408

[8] W. Li , U.‐H. Kim , A. Dolocan , Y.‐K. Sun , A. Manthiram , ACS Nano 2017, 11, 5853. 28502161 10.1021/acsnano.7b01494

[9] R. Jung , F. Linsenmann , R. Thomas , J. Wandt , S. Solchenbach , F. Maglia , C. Stinner , M. Tromp , H. A. Gasteiger , J. Electrochem. Soc. 2019, 166, A378.

[10] X. Zhang , Z. Cui , A. Manthiram , Adv. Energy Mater. 2022, 12, 2103611.

[11] A. Arifiadi , T. Brake , F. Demelash , B. Ying , K. Kleiner , H. Hur , S. Wiemers‐Meyer , M. Winter , J. Kasnatscheew , Adv. Energy. Sustain. Res. 2024.

[12] M. Dubarry , C. Truchot , B. Y. Liaw , K. Gering , S. Sazhin , D. Jamison , C. Michelbacher , J. Power Sources 2011, 196, 10336.

[13] J. C. Burns , A. Kassam , N. N. Sinha , L. E. Downie , L. Solnickova , B. M. Way , J. R. Dahn , J. Electrochem. Soc. 2013, 160, A1451.

[14] X. Ma , J. E. Harlow , J. Li , L. Ma , D. S. Hall , S. Buteau , M. Genovese , M. Cormier , J. R. Dahn , J. Electrochem. Soc. 2019, 166, A711.

[15] A. Arifiadi , F. Demelash , T. Brake , C. Lechtenfeld , S. Klein , L. Alsheimer , S. Wiemers‐Meyer , M. Winter , J. Kasnatscheew , Energy & Environ Materials 2024.

[16] D. S. Hall , T. Hynes , C. P. Aiken , J. R. Dahn , J. Electrochem. Soc. 2020, 167, 100538.

[17] S. Klein , P. Harte , S. van Wickeren , K. Borzutzki , S. Röser , P. Bärmann , S. Nowak , M. Winter , T. Placke , J. Kasnatscheew , Cell Rep. Phys. Sci. 2021, 2, 100521.

[18] S. Klein , L. Haneke , P. Harte , L. Stolz , S. van Wickeren , K. Borzutzki , S. Nowak , M. Winter , T. Placke , J. Kasnatscheew , ChemElectroChem 2022, 9.

[19] C. Wang , L. Yu , W. Fan , J. Liu , L. Ouyang , L. Yang , M. Zhu , ACS Appl. Energy Mater. 2018, 1, 2647.

[20] S. Klein , P. Bärmann , O. Fromm , K. Borzutzki , J. Reiter , Q. Fan , M. Winter , T. Placke , J. Kasnatscheew , J. Mater. Chem. A 2021, 9, 7546.

[21] M. Yoon , Y. Dong , J. Hwang , J. Sung , H. Cha , K. Ahn , Y. Huang , S. J. Kang , J. Li , J. Cho , Nat. Energy 2021, 6, 362.

[22] J. Chen , H. Chen , Y. Mei , J. Gao , A. Dai , Y. Tian , W. Deng , G. Zou , H. Hou , C. E. Banks , T. Liu , K. Amine , X. Ji , Energy Storage Mater. 2022, 52, 736.

[23] C.‐C. Su , M. He , P. C. Redfern , L. A. Curtiss , I. A. Shkrob , Z. Zhang , Energy Environ. Sci. 2017, 10, 900.

[24] Z. Zhang , L. Hu , H. Wu , W. Weng , M. Koh , P. C. Redfern , L. A. Curtiss , K. Amine , Energy Environ. Sci. 2013, 6, 1806.

[25] C.‐C. Su , M. He , J. Shi , R. Amine , Z. Yu , L. Cheng , J. Guo , K. Amine , Energy Environ. Sci. 2021, 14, 3029.

[26] F. Demelash , A. Gomez‐Martin , B. Heidrich , E. Adhitama , P. Harte , A. Javed , A. Arifiadi , M. M. Bela , P. Yan , D. Diddens , M. Winter , P. Niehoff , Small Struct. 2024.

[27] D. R. Gallus , R. Schmitz , R. Wagner , B. Hoffmann , S. Nowak , I. Cekic‐Laskovic , R. W. Schmitz , M. Winter , Electrochim. Acta 2014, 134, 393.

[28] L. D. Ellis , I. G. Hill , K. L. Gering , J. R. Dahn , J. Electrochem. Soc. 2017, 164, A2426.

[29] Z.‐B. Zhou , M. Takeda , T. Fujii , M. Ue , J. Electrochem. Soc. 2005, 152, A351.

[30] L. D. Ellis , J. Xia , A. J. Louli , J. R. Dahn , J. Electrochem. Soc. 2016, 163, A1686.

[31] L. Nyholm , T. Ericson , A. S. Etman , Chem. Eng. Sci. 2023, 282, 119346.

[32] S. Klein , S. van Wickeren , S. Röser , P. Bärmann , K. Borzutzki , B. Heidrich , M. Börner , M. Winter , T. Placke , J. Kasnatscheew , Adv. Energy Mater. 2021, 11, 2003738.

[33] A. Ghaur , C. Peschel , I. Dienwiebel , L. Haneke , L. Du , L. Profanter , A. Gomez‐Martin , M. Winter , S. Nowak , T. Placke , Adv. Energy Mater. 2023, 13, 2203503.

[34] J.‐P. Schmiegel , R. Nölle , J. Henschel , L. Quach , S. Nowak , M. Winter , F. Glorius , T. Placke , Cell Rep. Phys. Sci. 2021, 2, 100327.

[35] M. Weiling , C. Lechtenfeld , F. Pfeiffer , L. Frankenstein , D. Diddens , J.‐F. Wang , S. Nowak , M. Baghernejad , Adv. Energy Mater. 2024, 14, 2470022.

[36] J. Xia , R. Petibon , D. Xiong , L. Ma , J. R. Dahn , J. Power Sources 2016, 328, 124.

[37] C. C. Nguyen , B. L. Lucht , J. Electrochem. Soc. 2018, 165, A2154.

[38] Z. Chen , J. Liu , K. Amine , Electrochem. Solid‐State Lett. 2007, 10, A45.

[39] A. J. Smith , J. C. Burns , D. Xiong , J. R. Dahn , J. Electrochem. Soc. 2011, 158, A1136

[40] B. Streipert , L. Stolz , G. Homann , P. Janßen , I. Cekic‐Laskovic , M. Winter , J. Kasnatscheew , ChemSusChem 2020, 13, 5301. 32692891 10.1002/cssc.202001530 PMC7589409

[41] J. Kasnatscheew , B. Streipert , S. Röser , R. Wagner , I. Cekic Laskovic , M. Winter , Phys. Chem. Chem. Phys. 2017, 19, 16078. 28597888 10.1039/c7cp03072j

[42] A. Gomez‐Martin , M. M. Gnutzmann , E. Adhitama , L. Frankenstein , B. Heidrich , M. Winter , T. Placke , Adv. Sci. (Weinheim, Baden‐Wurttemberg, Germany) 2022, 9, 2201742. 10.1002/advs.202201742 PMC9403639 35798310

[43] B. S. Parimalam , B. L. Lucht , J. Electrochem. Soc. 2018, 165, A251.

[44] S. Jurng , Z. L. Brown , J. Kim , B. L. Lucht , Energy Environ. Sci. 2018, 11, 2600.

[45] J. Kasnatscheew , U. Rodehorst , B. Streipert , S. Wiemers‐Meyer , R. Jakelski , R. Wagner , I. C. Laskovic , M. Winter , J. Electrochem. Soc. 2016, 163, A2943.

[46] Q. Dong , F. Guo , Z. Cheng , Y. Mao , R. Huang , F. Li , H. Dong , Q. Zhang , W. Li , H. Chen , Z. Luo , Y. Shen , X. Wu , L. Chen , ACS Appl. Energy Mater. 2020, 3, 695.

[47] M. Kubot , L. Frankenstein , E. Muschiol , S. Klein , M. Esselen , M. Winter , S. Nowak , J. Kasnatscheew , ChemSusChem 2023, 16, 202202189. 10.1002/cssc.202202189 36533855

[48] V. Meunier , M. Leal De Souza , M. Morcrette , A. Grimaud , Joule 2023, 7, 42.

[49] G. Homann , L. Stolz , K. Neuhaus , M. Winter , J. Kasnatscheew , Adv. Funct. Mater. 2020, 30, 2006289.

[50] A. J. Sanchez , E. Kazyak , Y. Chen , K.‐H. Chen , E. R. Pattison , N. P. Dasgupta , ACS Energy Lett. 2020, 5, 994.

[51] A. B. Gunnarsdóttir , C. V. Amanchukwu , S. Menkin , C. P. Grey , J. Am. Chem. Soc. 2020, 142, 20814. 33226793 10.1021/jacs.0c10258 PMC7729915

[52] P. Yan , M. Shevchuk , C. Wölke , F. Pfeiffer , D. Berghus , M. Baghernejad , G.‐V. Röschenthaler , M. Winter , I. Cekic‐Laskovic , Small Struct. 2023, 5, 2300425.

[53] E. Trevisanello , R. Ruess , G. Conforto , F. H. Richter , J. Janek , Adv. Energy Mater. 2021, 11, 2003400.

[54] X. Min , C. Han , S. Zhang , J. Ma , N. Hu , J. Li , X. Du , B. Xie , H.‐J. Lin , C.‐Y. Kuo , C.‐T. Chen , Z. Hu , L. Qiao , Z. Cui , G. Xu , G. Cui , Angew. Chem., Int. Ed. 2023, 62, 202302664. 10.1002/anie.202302664 37349889

[55] I. A. Shkrob , Y. Zhu , T. W. Marin , D. P. Abraham , J. Phys. Chem. C 2013, 117, 23750.

[56] W. M. Dose , W. Li , I. Temprano , C. A. O'Keefe , B. L. Mehdi , M. F. L. de Volder , C. P. Grey , ACS Energy Lett. 2022, 7, 3524. 36277132 10.1021/acsenergylett.2c01722 PMC9578037

[57] C. Peebles , R. Sahore , J. A. Gilbert , J. C. Garcia , A. Tornheim , J. Bareño , H. Iddir , C. Liao , D. P. Abraham , J. Electrochem. Soc. 2017, 164, A1579.

[58] A. J. Louli , A. Eldesoky , R. Weber , M. Genovese , M. Coon , J. deGooyer , Z. Deng , R. T. White , J. Lee , T. Rodgers , R. Petibon , S. Hy , S. J. H. Cheng , J. R. Dahn , Nat. Energy 2020, 5, 693.

[59] R. Weber , M. Genovese , A. J. Louli , S. Hames , C. Martin , I. G. Hill , J. R. Dahn , Nat. Energy 2019, 4, 683.

[60] Y. Zhu , Y. Li , M. Bettge , D. P. Abraham , J. Electrochem. Soc. 2012, 159, A2109.

[61] S. Di Muzio , O. Palumbo , S. Brutti , A. Paolone , J. Electrochem. Soc. 2022, 169, 070523.

[62] P. Gupta , P. Gudmundson , J. Power Sources 2023, 582, 233514.

[63] T. Schwieters , M. Evertz , A. Fengler , M. Börner , T. Dagger , Y. Stenzel , P. Harte , M. Winter , S. Nowak , J. Power Sources 2018, 380, 194.

[64] S. Hwang , D.‐H. Kim , J. H. Shin , J. E. Jang , K. H. Ahn , C. Lee , H. Lee , J. Phys. Chem. C 2018, 122, 19438.

[65] S. S. Zhang , K. Xu , T. R. Jow , J. Electrochem. Soc. 2002, 149, A586.

[66] L. Stolz , S. Röser , G. Homann , M. Winter , J. Kasnatscheew , J. Phys. Chem. C 2021, 125, 18089.

[67] R. Sahore , D. C. O'Hanlon , A. Tornheim , C.‐W. Lee , J. C. Garcia , H. Iddir , M. Balasubramanian , I. Bloom , J. Electrochem. Soc. 2020, 167, 020513.

[68] H. J. Chang , N. M. Trease , A. J. Ilott , D. Zeng , L.‐S. Du , A. Jerschow , C. P. Grey , J. Phys. Chem. C 2015, 119, 16443.

[69] E. Adhitama , F. Demelash , T. Brake , A. Arifiadi , M. Vahnstiege , A. Javed , M. Winter , S. Wiemers‐Meyer , T. Placke , Adv. Energy Mater. 2024, 14.

[70] A. Narayanan Krishnamoorthy , C. Wölke , D. Diddens , M. Maiti , Y. Mabrouk , P. Yan , M. Grünebaum , M. Winter , A. Heuer , I. Cekic‐Laskovic , Chem. Methods 2022, 2.

[71] B. Vortmann‐Westhoven , M. Winter , S. Nowak , J. Power Sources 2017, 346, 63.

[72] M. Evertz , J. Kasnatscheew , M. Winter , S. Nowak , Anal. Bioanal. Chem. 2019, 411, 277. 30374724 10.1007/s00216-018-1441-8

[73] T. O'Haver , peakfit.m, https://www.mathworks.com/matlabcentral/fileexchange/23611‐peakfit‐m (accessed: February 2024).

