# Supplementary material for: Evaluation of Alternative Lithium Salts for High‐Voltage Lithium Ion Batteries: Higher Relevance of Plated Li Morphology Than the Amount of Electrode Crosstalk
Source: Small. 2024 Dec 15;21(19):2410762. doi: 10.1002/smll.202410762 (PMC12067173; doi:10.1002/smll.202410762)
Supplement: Supplementary file 1 — Supporting Information [file SMLL-21-2410762-s001.docx]

**Supporting Information**

**Evaluation of Alternative Lithium Salts for High-Voltage Lithium Ion Batteries: Higher Relevance of Plated Li Morphology than the Amount of Electrode Crosstalk**

Anindityo Arifiadi^1,2^, Lennart Wichmann^3^, Tobias Brake^1^_,_ Christian Lechtenfeld^1^_,_ Julius Buchmann^1,2^, Feleke Demelash^1^, P. Yan^3^, Gunther Brunklaus^3^, I. Cekic-Laskovic^3^, Simon Wiemers-Meyer^1^, Martin Winter^1,3^, Johannes Kasnatscheew^1,*^

A. Arifiadi, T. Brake, C. Lechtenfeld, J. Buchmann , F. Demelash, Dr. S. Wiemers-Meyer, Prof. M. Winter, Dr. J. Kasnatscheew^*^

^1^ MEET Battery Research Center, Institute of Physical Chemistry, University of Münster

Corrensstr. 46, 48149 Münster, Germany

*E-mail: johannes.kasnatscheew@uni-muenster.de

*A. Arifiadi, J. Buchmann*

^2^ International Graduate School for Battery Chemistry, Characterization, Analysis, Recycling and Application (BACCARA), University of Münster

Corrensstr. 40, 48149 Münster, Germany

*L. Wichmann, P. Yan, PD Dr. G. Brunklaus, Dr. I. Cekic-Laskovic, Prof. Dr. M. Winter*

^3^ Helmholtz Institute Münster, IEK-12, Forschungszentrum Jülich GmbH

Corrensstr. 46, 48149 Münster, Germany

**Supplementary Note 1**

The NMR shift of lithium metal deposits is strongly influenced by their orientation to the magnetic field, with vertically oriented deposits displaying higher chemical shift than horizontally oriented deposits.^[1]^ This orientation-dependent chemical shift can be used to differentiate dendritic, mossy, and compact lithium deposits.^[2]^ Given the inhomogeneity, *i.e.,* variance in orientation, of lithium metal deposition often observed in Li ion batteries, the NMR spectra is made up of multiple peaks with multiple chemical shifts. In this work, the range of peak position representing compact, mossy, and dendritic lithium are based on model experiments supported by simulation results.^[2]^ It is possible to improve the fitting quality of the NMR spectra by increasing the degrees of freedom such as peak number, width and peak position range. However, to maintain realistic fitting parameters, only chemical shifts from previously reported lithium morphologies and peak width obtained from as-manufactured, compact lithium metal electrodes were considered.^[2]^

It is aslo notable that regardless of the peak fitting, the NMR spectra for electrodes cycled with LiPF_6_ clearly exhibit higher chemical shifts, supporting the dendritic microstructure observed in SEM images. Moreover, the spectra peak of the sample with LiBF_4_ shows lower chemical shift than that with LiDFOB, again supporting the more compact lithium deposit morphology of the former observed in SEM images. Fitting the NMR spectra with three voigt functions to decipher the contribution of different lithium species to the overall morphology adds a quantitative interpretation of the previously described qualitative SEM comparison, substantiating the observed differences between samples.


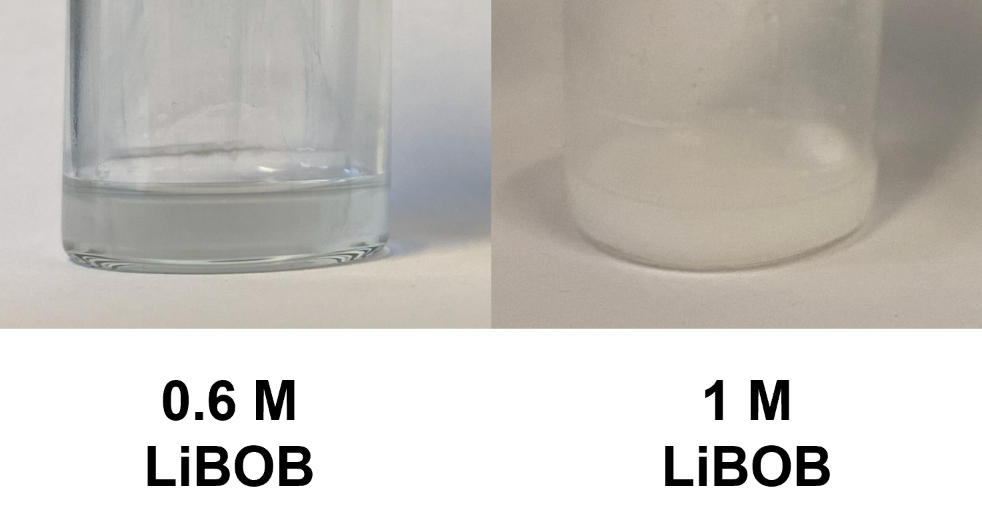


**Figure S1.** Photographs of 0.6 and 1 M LiBOB in an ethylene carbonate (EC)‑ethyl methyl carbonate (EMC) mixture (3:7 by weight). Note that an almost clear solution is achieved by lowering the LiBOB concentration to 0.6 M close to the solubility limit.

**
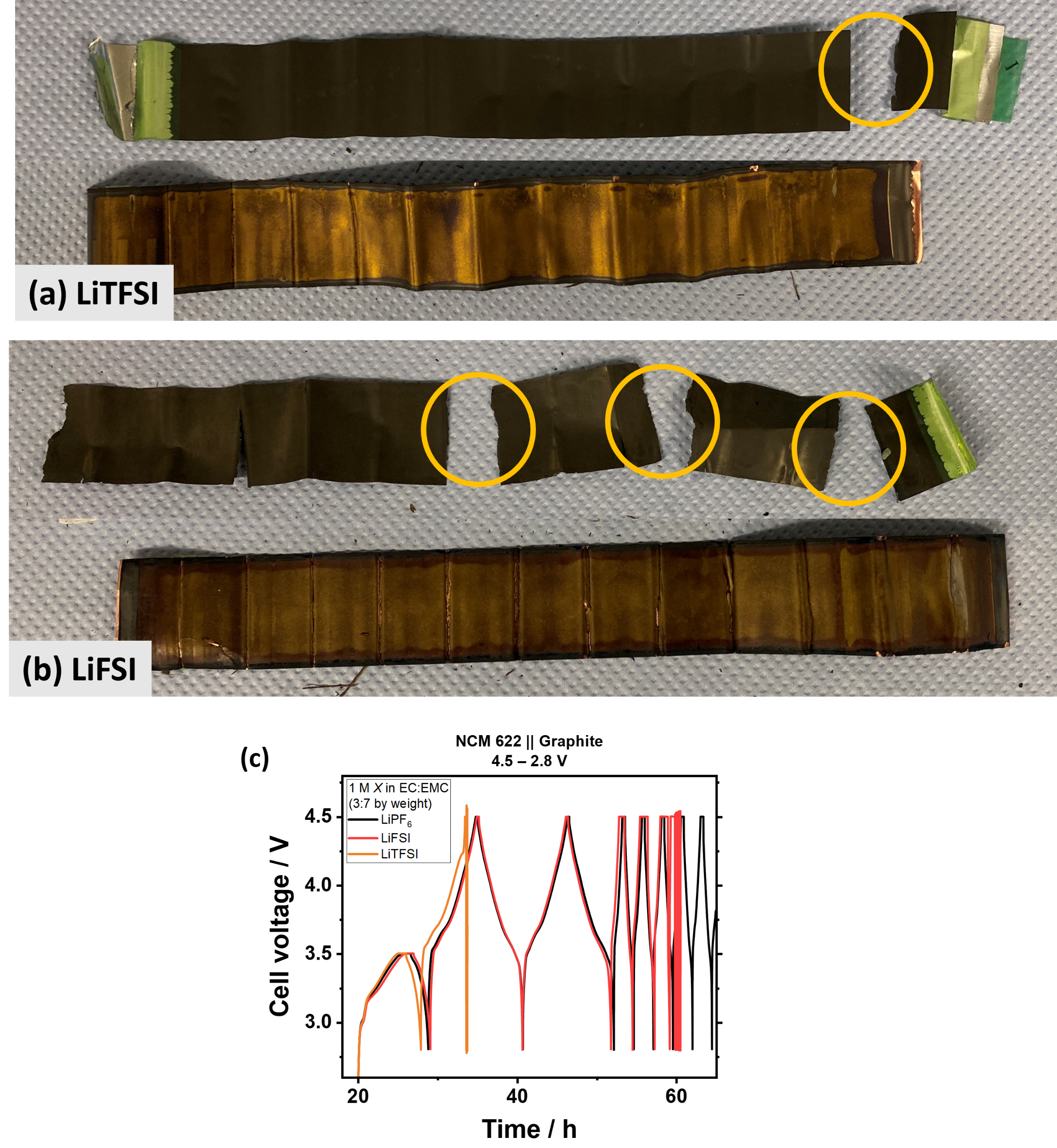
Figure S2.** Photographs of positive and negative electrodes extracted from cells with electrolytes containing (a) LiTFSI and (b) LiFSI salts, obtained after 102 charge/discharge cycles. The yellow circles in (a) and (b) indicate the areas with aluminium current collector disintegration, which can be attributed to aluminium dissolution. (c) Voltage profiles of cells with electrolytes using LiPF_6_, LiTFSI, and LiFSI salts in the first 65 hours. The lack of any discharge process in the cell with LiTFSI results into the fact graphite that cannot be delithiated, supported by the golden color of lithiated graphite in photograph (a).


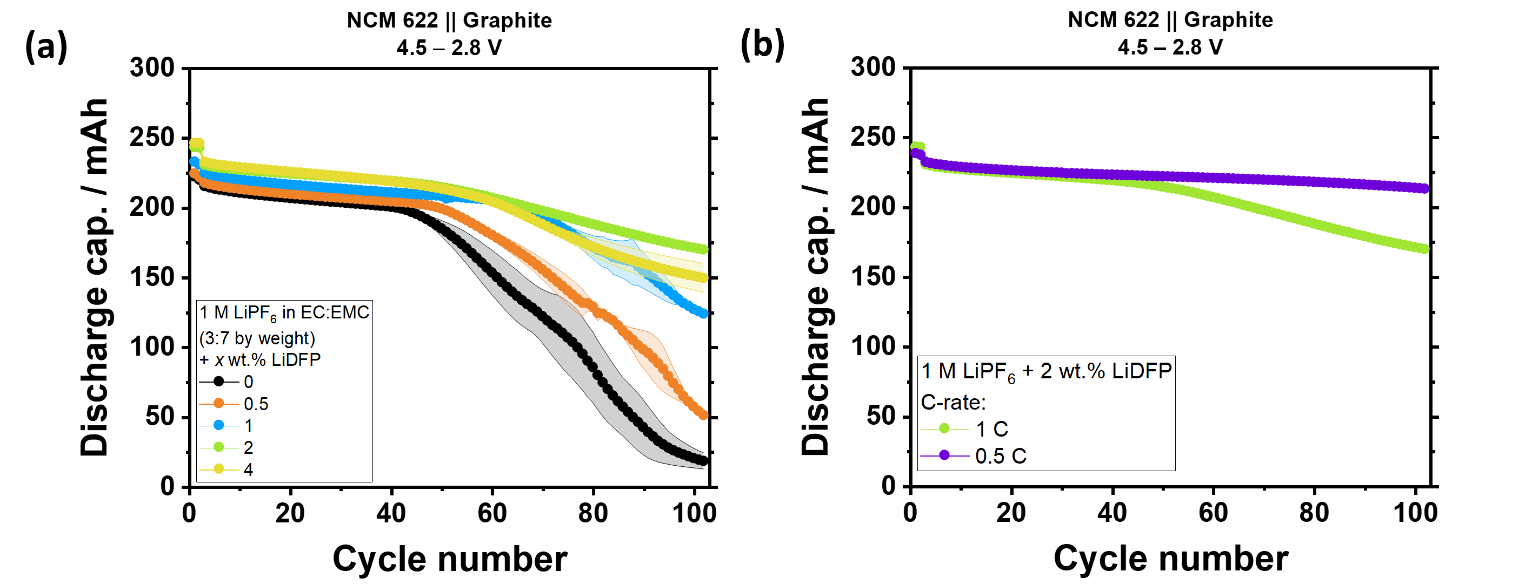


**Figure S3.** Discharge capacity *vs* cycle number plot of cells (a) with varied amounts of LiDFP and (b) with 2 wt.% LiDFP cycled at 0.5 and 1 C.


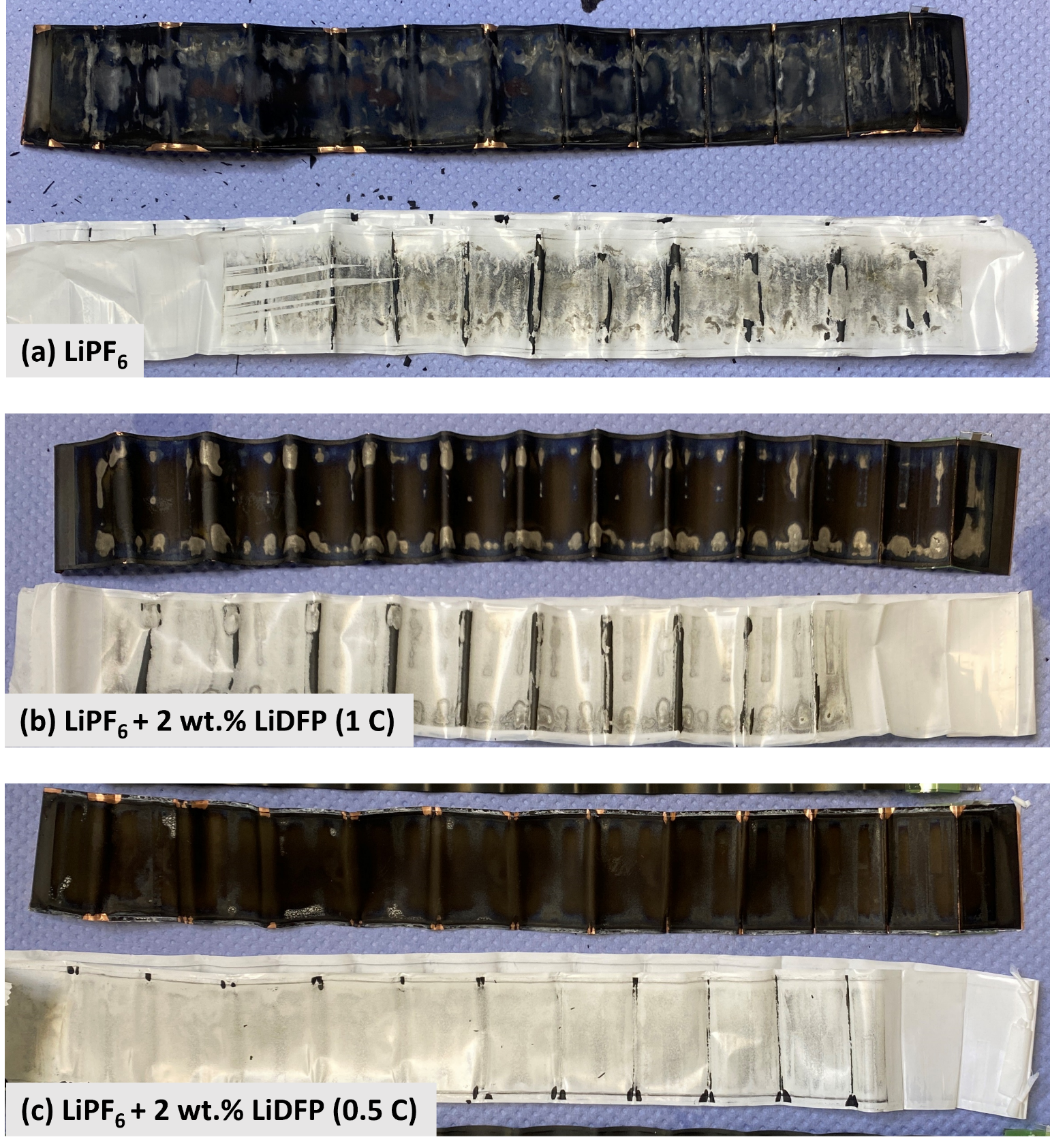


**Figure S4.** Photographs of negative electrode and separator obtained from cells with electrolytes using 1 M LiPF_6_ (a) electrolytes without LiDFP cycled at 1 C, (b) with 2 wt.% LiDFP cycled at 1 C, and (c) electrolytes with 2 wt.% LiDFP cycled at 0.5 C after 102 charge/discharge cycles.


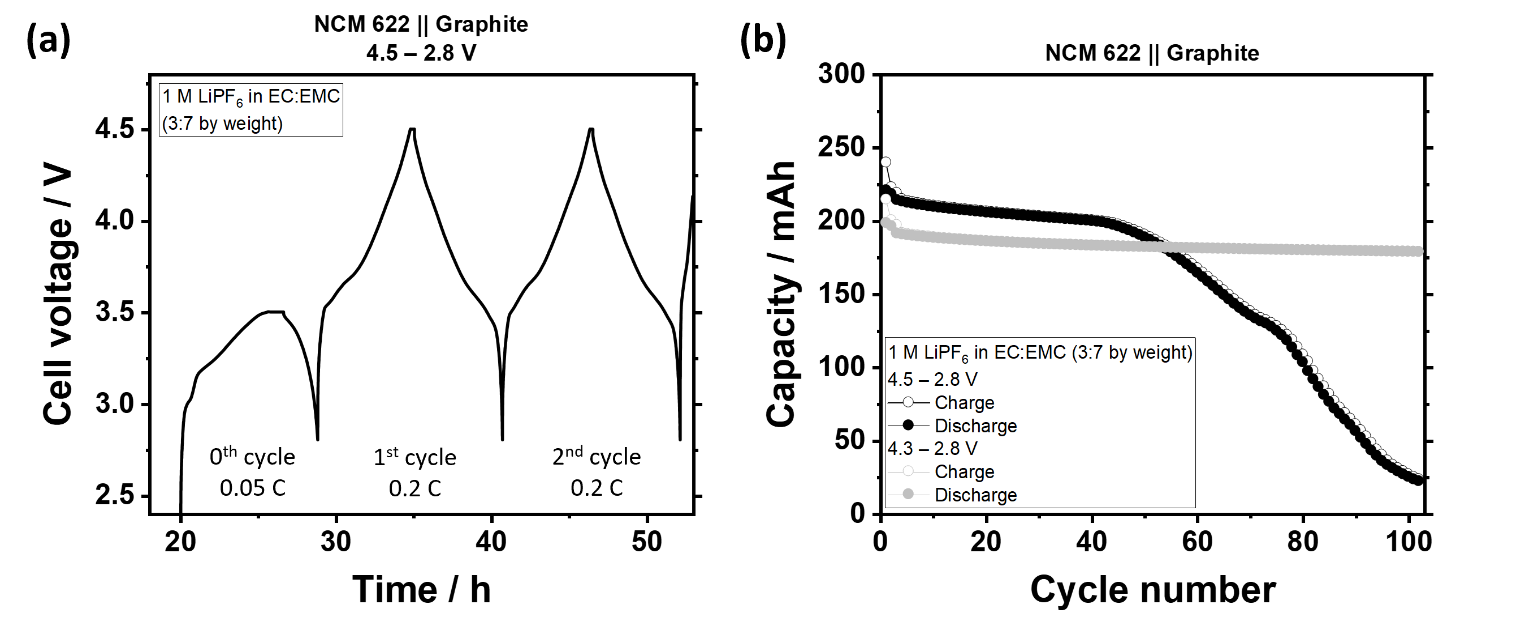


**Figure S5.** (a) Exemplary formation cycles voltage profile consisting of a pre‑charge (0^th^) cycle and two subsequent full‑charge cycles. (b) Charge/discharge capacity *vs.* cycle number of cells with LiPF_6_ salt charged to 4.3 and 4.5 V. Note that anomalous capacities are not observed during rollover fading.


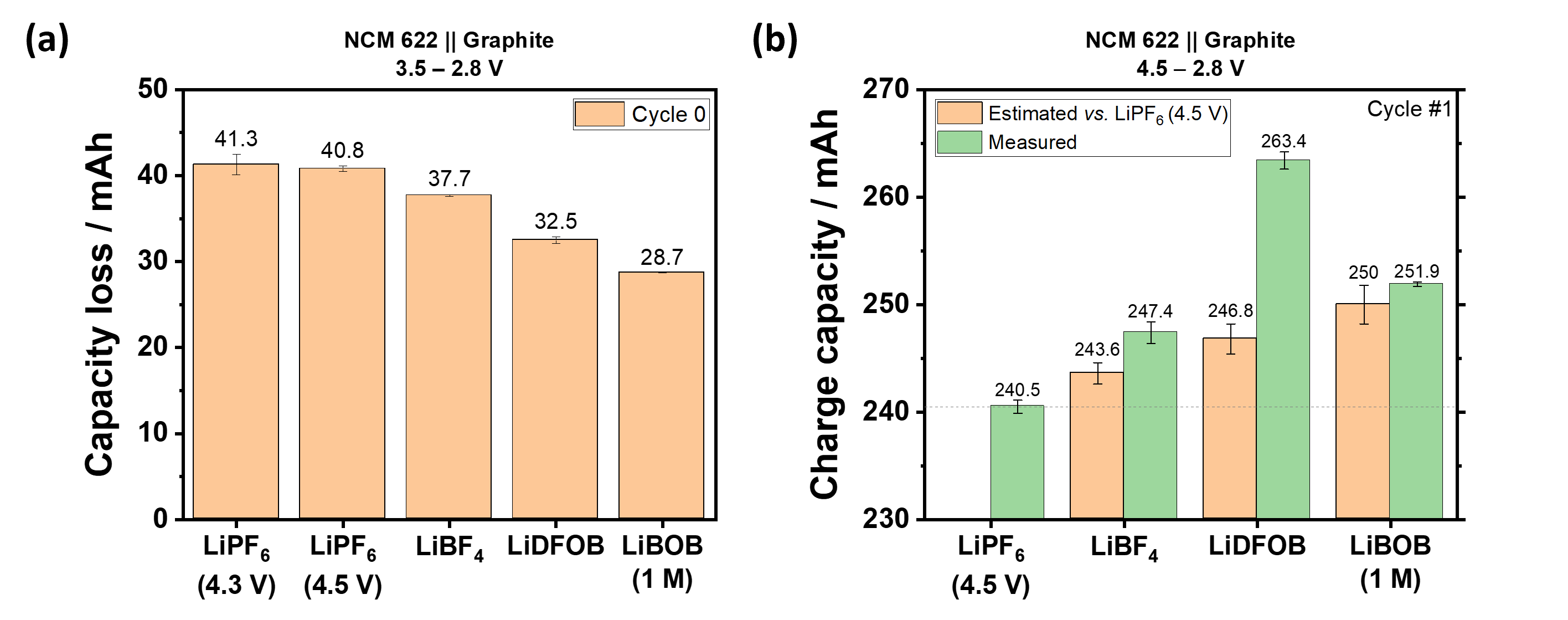


**Figure S6.** (a) Capacity loss in the 0^th^ and 1^st^ cycles and (e) estimated first cycle charge capacities.

In **Figure S6 (a)**, approximate Li loss values are obtained by subtracting the discharge capacity from the charge capacity in the respective cycle according to Equation (1). This is based on the assumption, that the capacity loss *Q*_loss_ is equal to the Li_loss_.

|  | $Li loss\approx Q_{loss}=Q_{\mathrm{charge}}-Q_{\mathrm{discharge}}$ | (1) |
| --- | --- | --- |

Subsequently, the differences in Li loss of cells with alternative salts *x* (*x* = LiBF_4_, LiDFOB, and LiBOB) *vs.* LiPF_6_ (ΔLi) at the 0^th^ cycle are calculated according to Equation (2).

|  | $\Delta\mathrm{Li}_{x}=Li \mathrm{loss}_{\mathrm{LiPF}_{6}}-Li \mathrm{loss}_{x}$ | (2) |
| --- | --- | --- |

To estimate the maximum charge capacities (*Q*_c(est)_) that can be reached by cells with alternative salts in the 1^st^ cycle (assuming no additional active lithium is introduced to the system, *e.g.,* from electrolyte oxidation), ΔLi of cells with alternative salts is added to the 0^th^ cycle charge capacity of cells with LiPF_6_, as seen in Equation (3).

|  | $Q_{c(es{t)}_{x}}=Q_{c_{\mathrm{LiPF}_{6}}}+\Delta\mathrm{Li}_{x}$ | (3) |
| --- | --- | --- |

Taking into account the difference in Li loss during SEI formation in cells with electrolytes containing LiBF_4_, LiDFOB, and LiBOB compared to LiPF_6_ (ΔLi, **Figure S6 (a)**), the maximum 1^st^ cycle charge capacities for cells with electrolytes containing LiBF_4_, LiDFOB, and LiBOB are estimated by adding ΔLi to the 1^st^ cycle charge capacities cells with LiPF_6_. As seen in **Figure S6 (b)**, the measured charge capacities of cells with electrolytes containing LiBOB are within the estimated value, whereas the measured capacities of cells with LiBF_4_ and LiDFOB are higher than the estimated values.


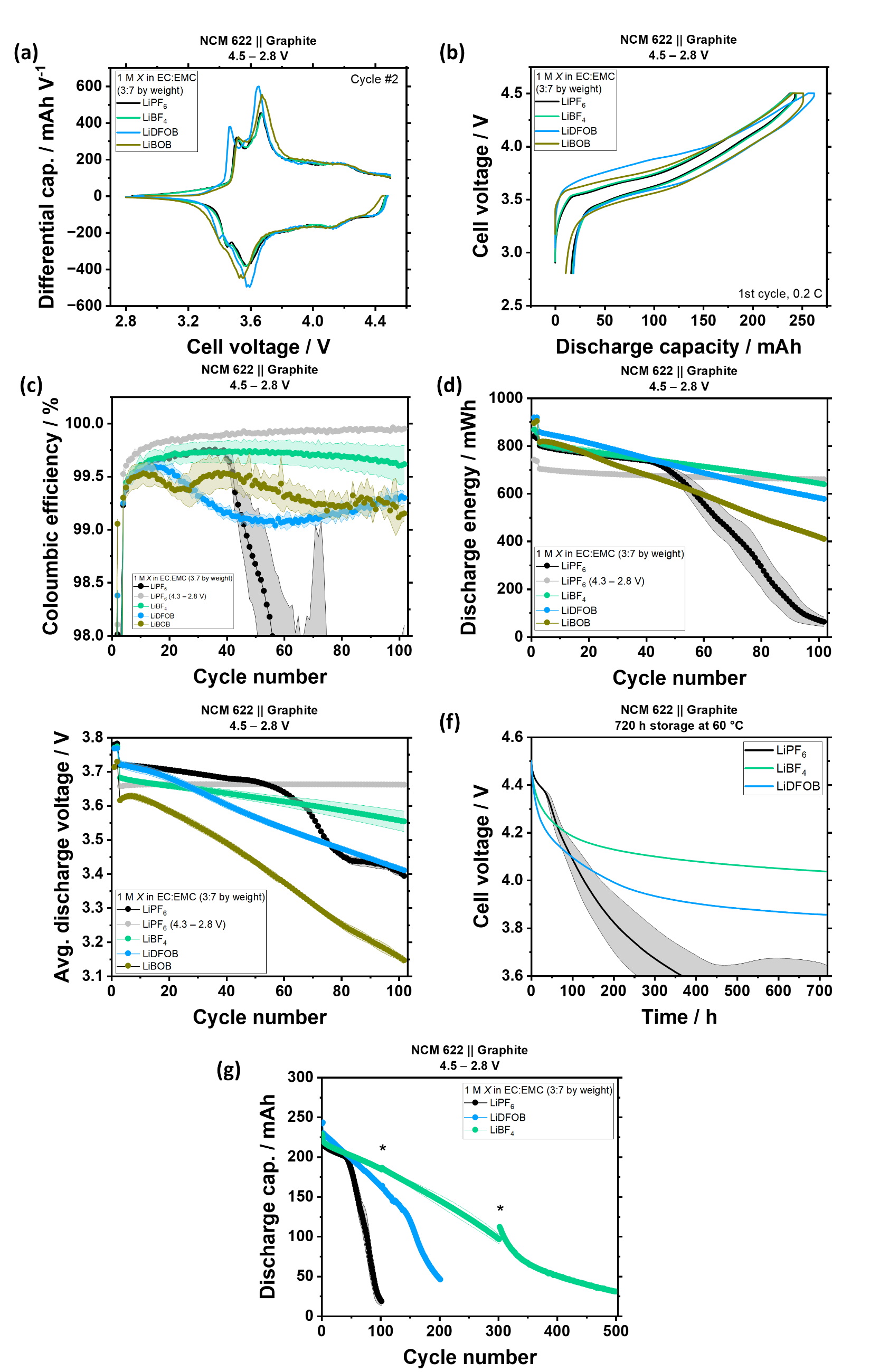


**Figure S7.** (a) Second cycle differential capacity *vs.* voltage profiles, (b) first cycle capacity *vs.* voltage profiles, (c) Couloumbic efficiency *vs.* cycle number plots, (d) average discharge voltage *vs.* cycle number plots, (e) discharge energy *vs.* cycle number plots, (f) cell voltage *vs.* time plots, and (g) discharge capacity *vs.* cycle number plots of cells with 1 M LiPF_6_, 1 M LiDFOB, and 1 M LiBF_4_ and.1 M LiBOB. The asterisks shown in (g) indicate cycles after degassing step.


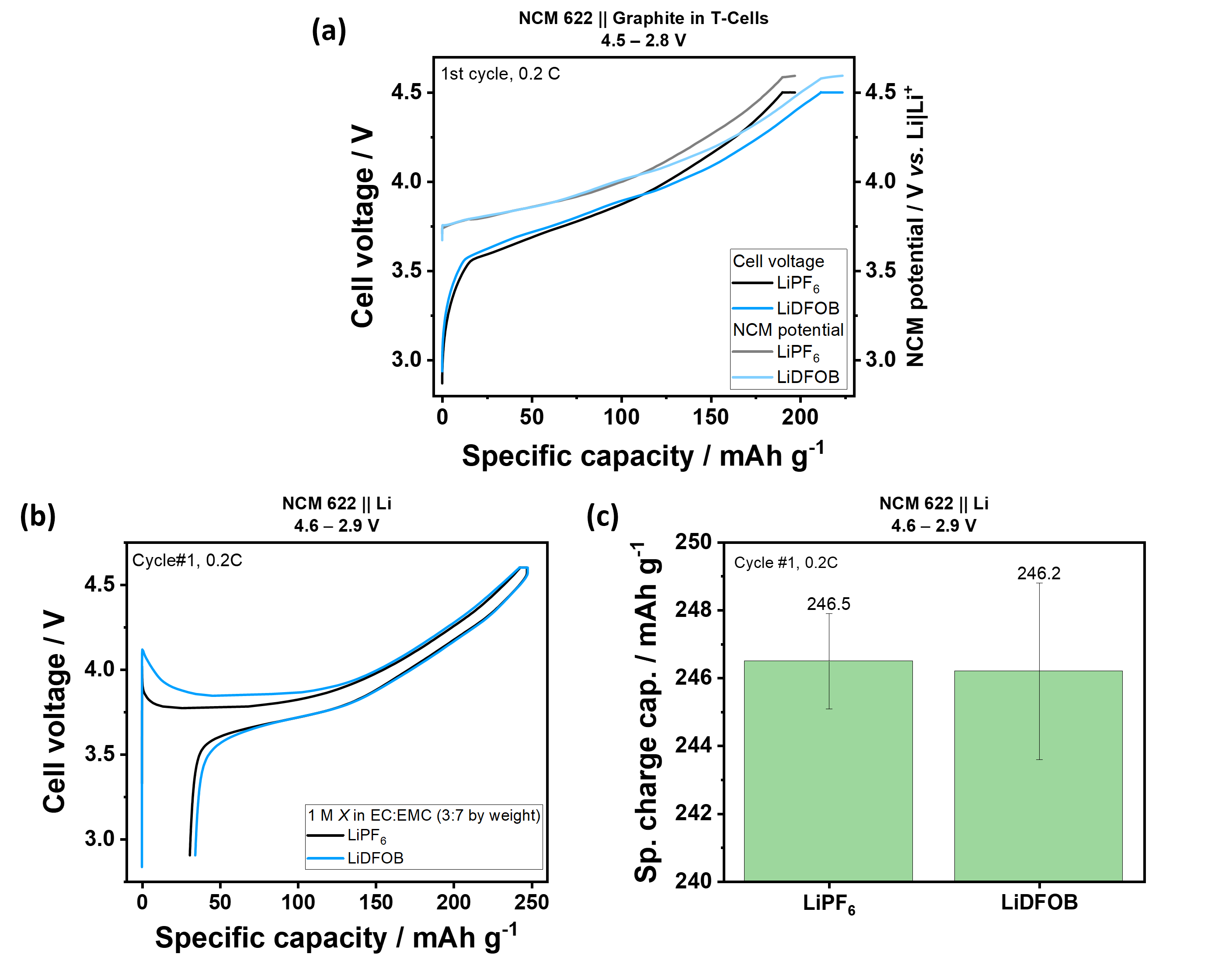


**Figure S8.** First cycle (a) cell voltage and NCM potential *vs.* capacity profiles in T-cells, (b) cell voltage *vs.* capacity profiles of NCM || Li cells, and (c) charge capacities of NCM 622 || Li cells with 1 M LiPF_6_,, 1 M LiDFOB electrolytes.


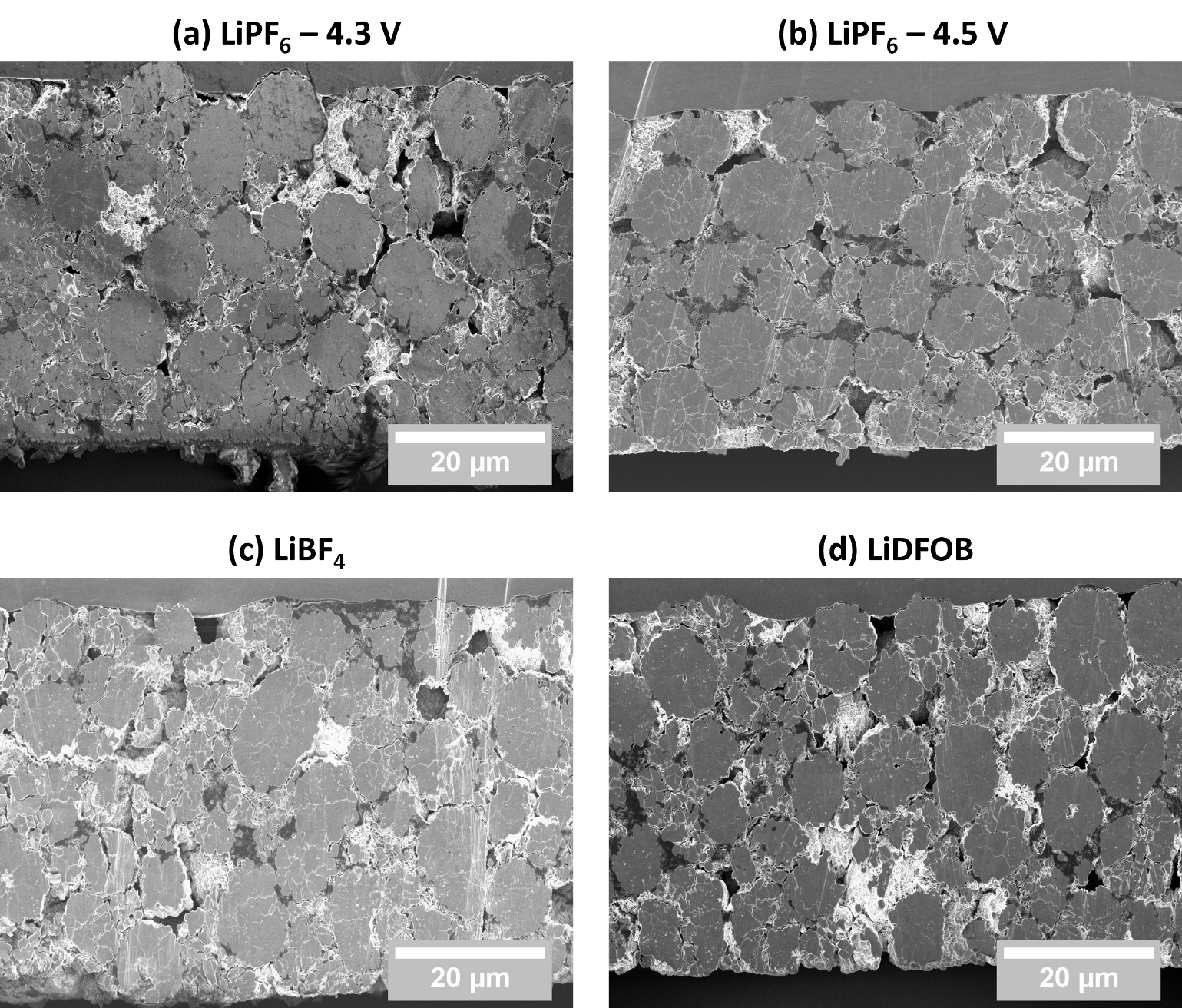


**Figure S9.** Cross section SEM images of positive electrodes after 102 charge/discharge cycled in cells with (a) LiPF_6_ (4.3 V UCV), (b) LiPF_6_ (4.5 V UCV), (c) LiBF_4_, (d) LiDFOB. Note that particle cracking is observed in (b)‑(d).


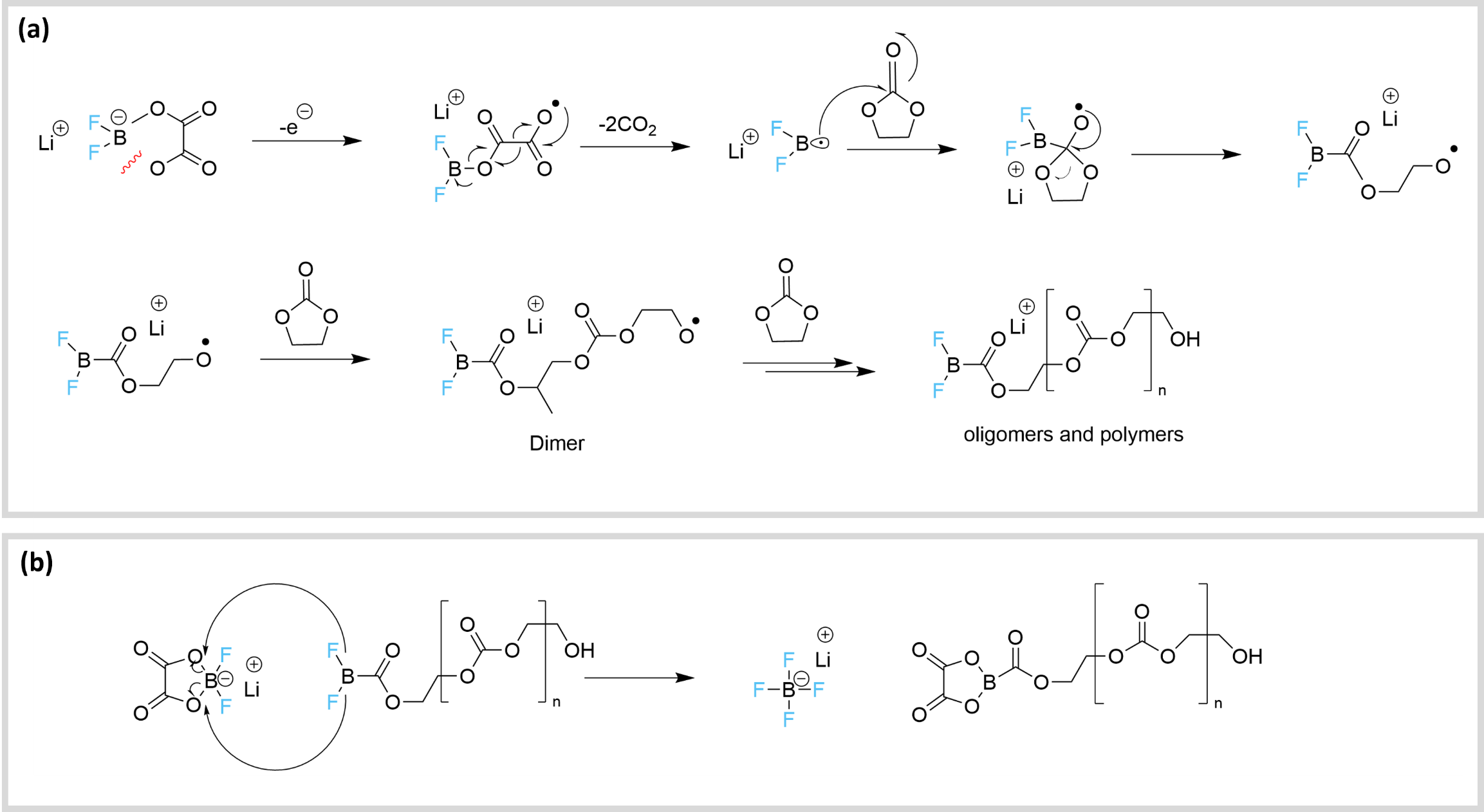


**Figure S10.** Proposed reaction mechanisms: (a) LiDFOB oxidation and its subsequent polymerization with EC.^[3]^ (b) LiBF_4_ formation from fluorine-oxygen exchange between LiDFOB and fluorinated boron center in the polymeric product in (a).^[4]^


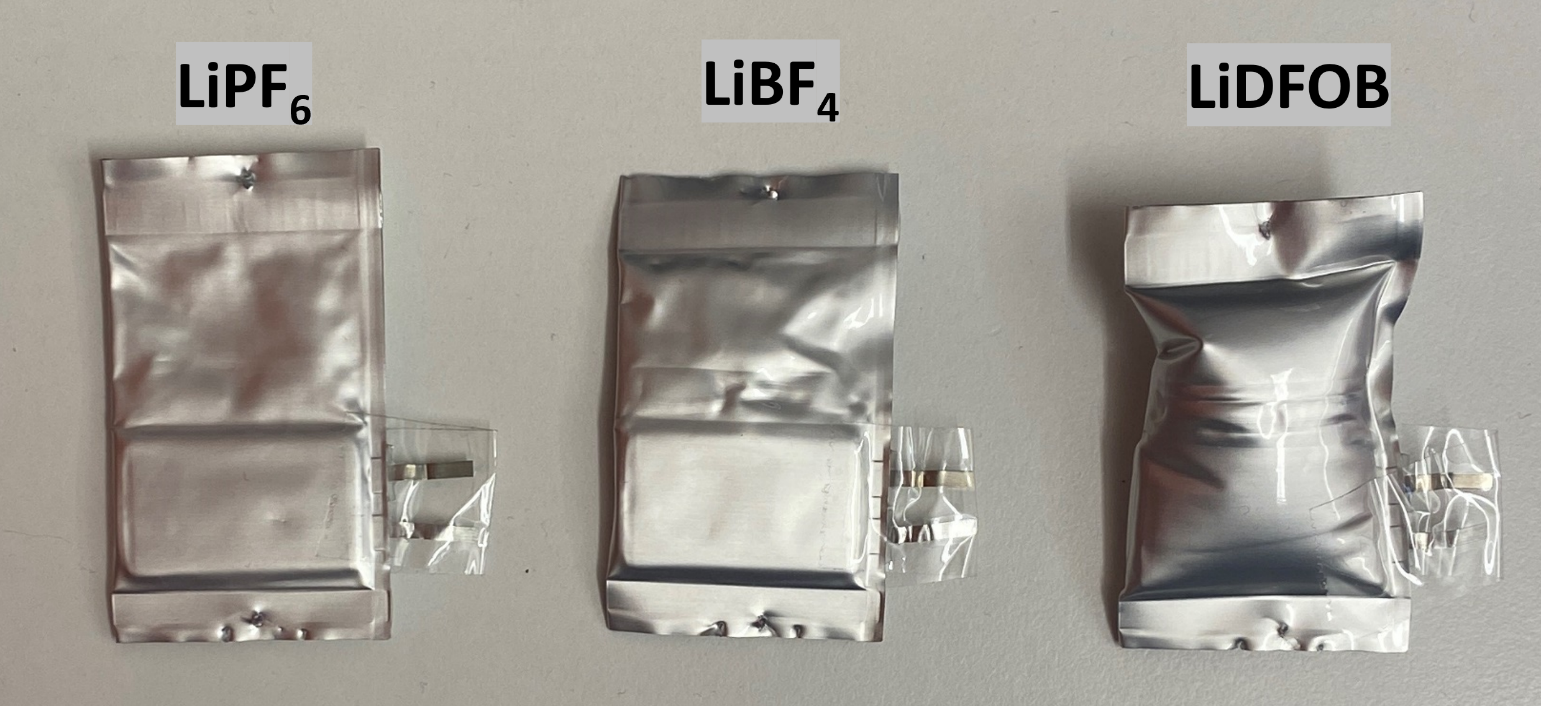


**Figure S11.** Photograph of NCM 622 || graphite pouch cells after 102 charge/discharge cycles (4.5 V UCV) with electrolytes containing LiPF_6_, LiBF_4_, and LiDFOB salts showing large gas production for the cell with LiDFOB electrolyte.


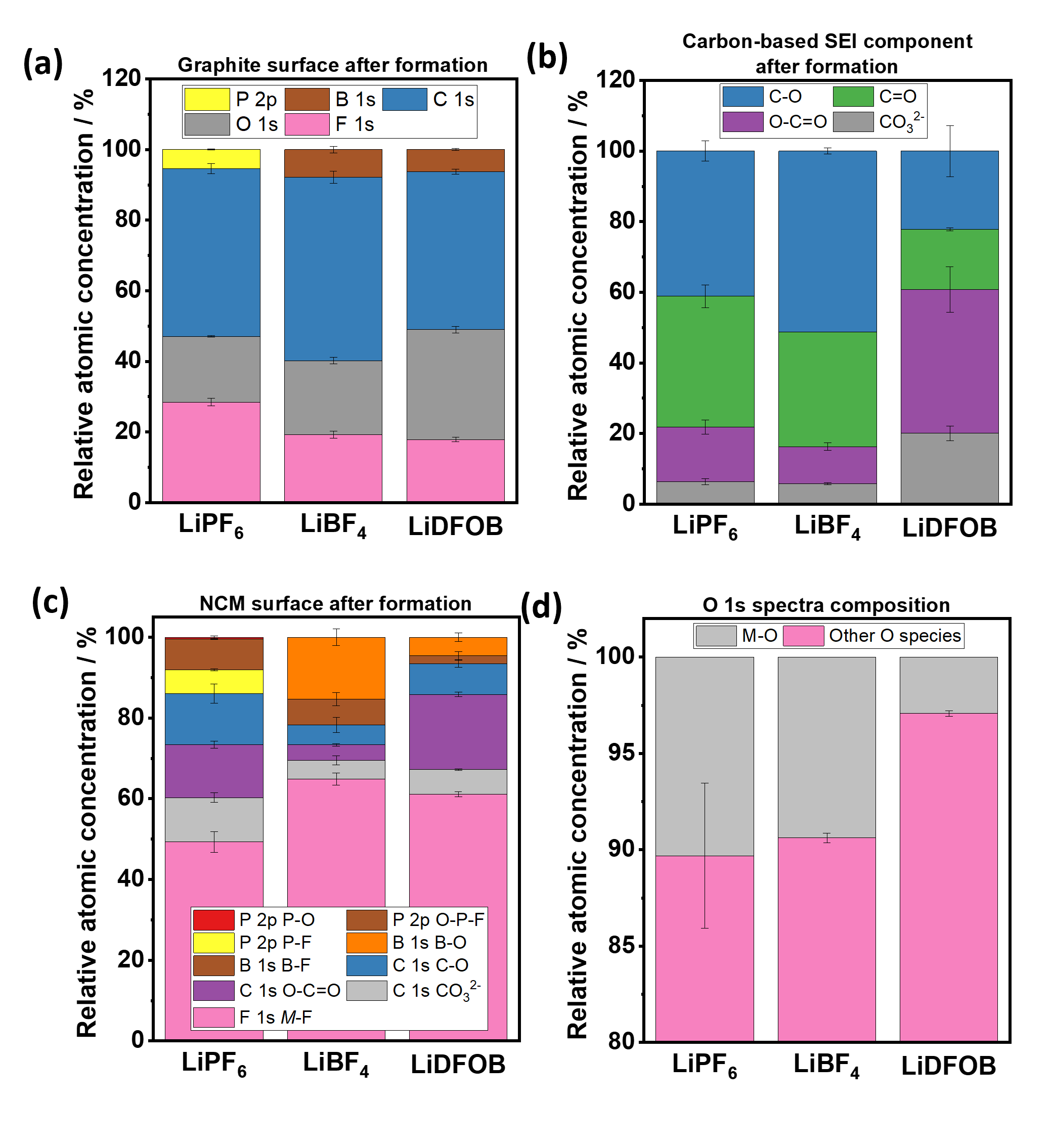


**Figure S12.** Surface composition of (a,b) graphite and (c,d) NCM 622 after formation cycles obtained *via* XPS. (b) Carbon-based SEI components from the C 1s spectra in (a), showing higher concentration of O-C=O species from lithium oxalate from DFOB^-^ reduction in the case of LiDFOB.^[5,6]^ (d) Ratio of M-O component compared to other O species in the O 1s spectra showing low concentration of M­O component in the case of LiDFOB, suggesting thicker CEI coverage.


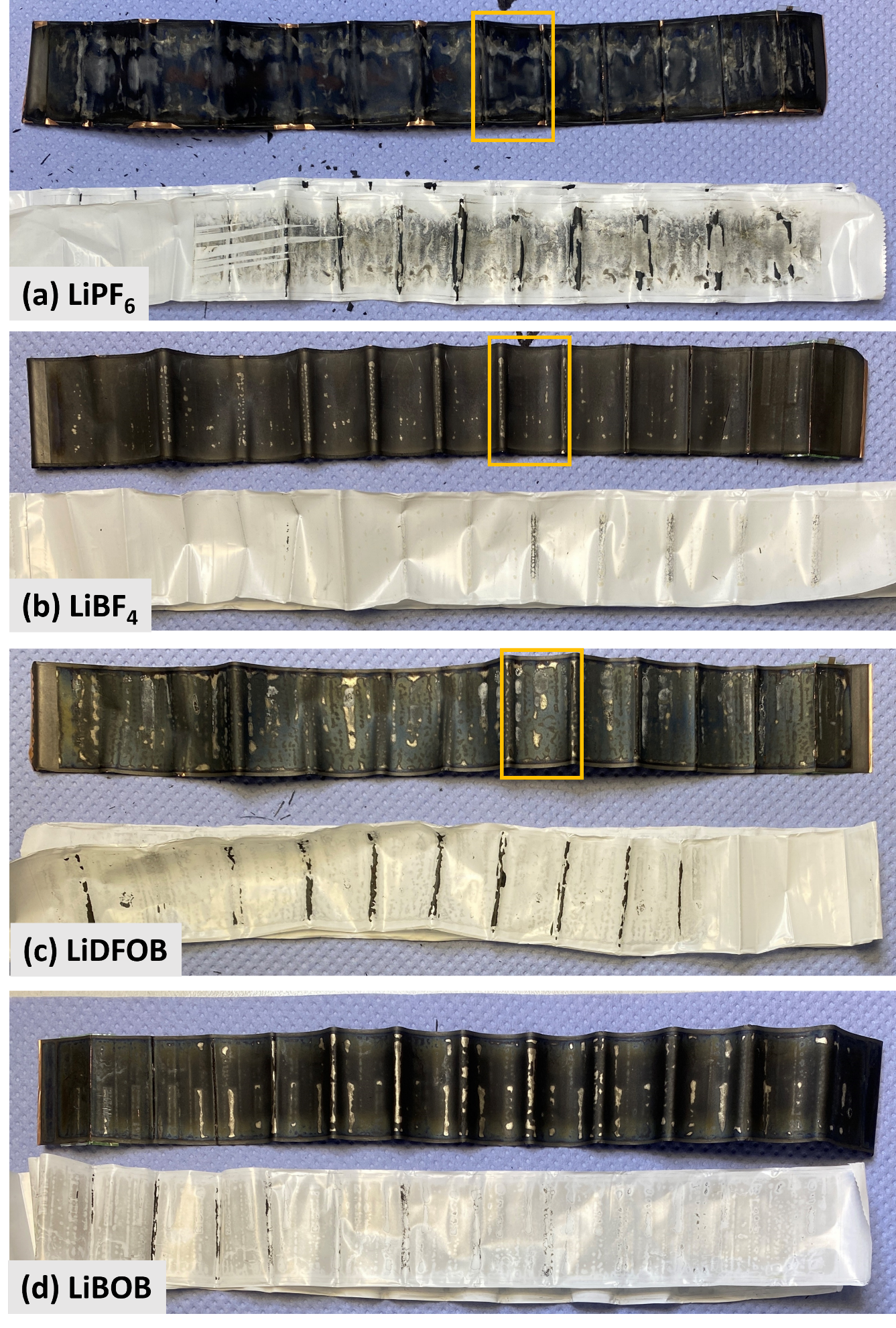


**Figure S13.** Photographs of negative electrode and separator obtained from cells with electrolytes containing (a) 1 M LiPF_6_, (b) 1 M LiBF_4_, (c) 1 M LiDFOB, and (d) 1 M LiBOB after 102 charge/discharge cycles.


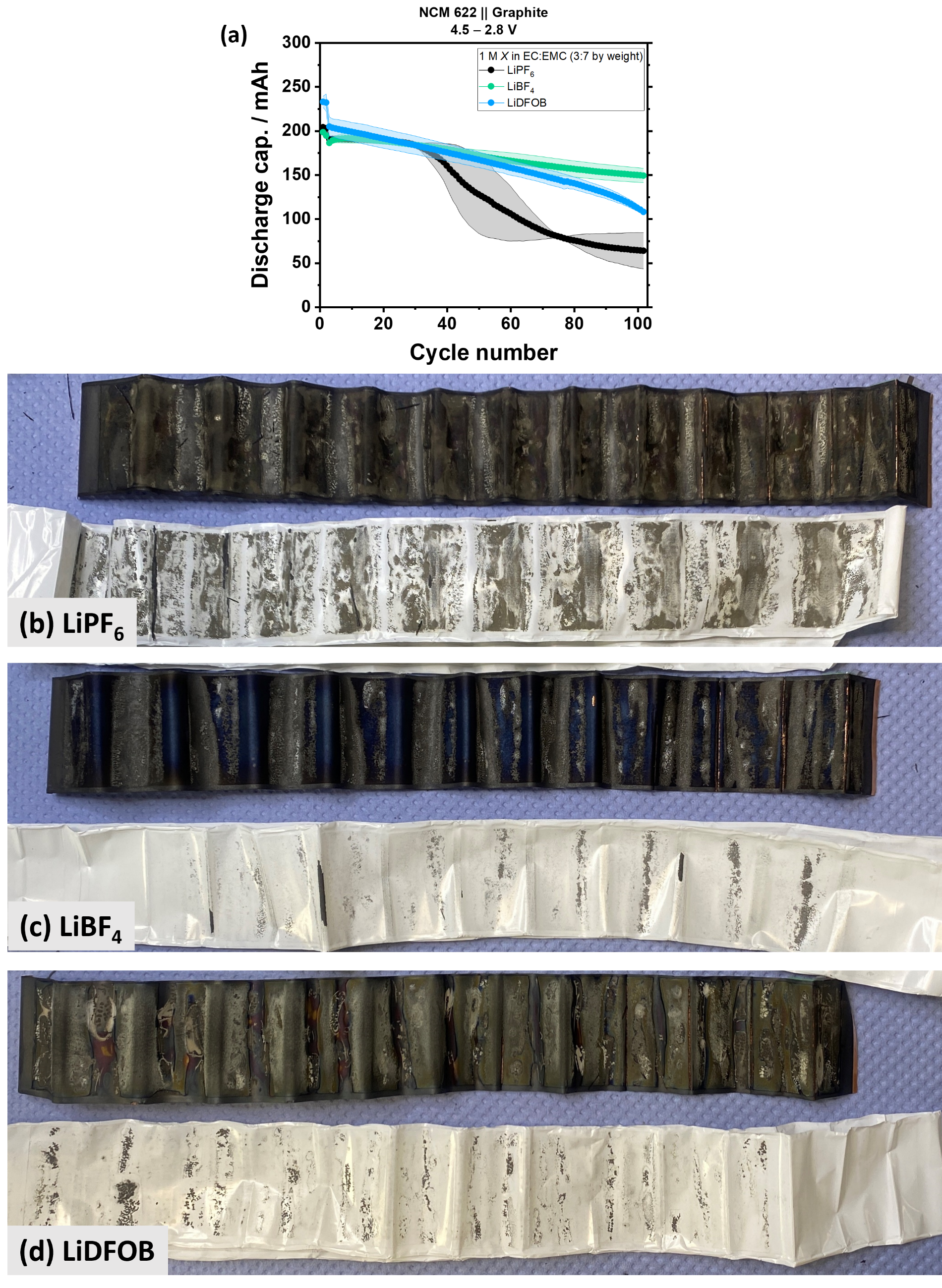


**Figure S14.** (a) Discharge capacity *vs.* cycle number plot of cells cycled without any applied pressure. Photographs of negative electrodes obtained from cells with electrolytes containing (b) LiPF_6_, (c) LiBF_4_, and (d) LiDFOB after 102 cycles without applied pressure. It is notable that separators from cells with electrolytes containing LiBF_4_, and LiDFOB have less black residue, indicating that less metallic lithium is penetrating the separator, *i.e,* less dendritic lithium plating.


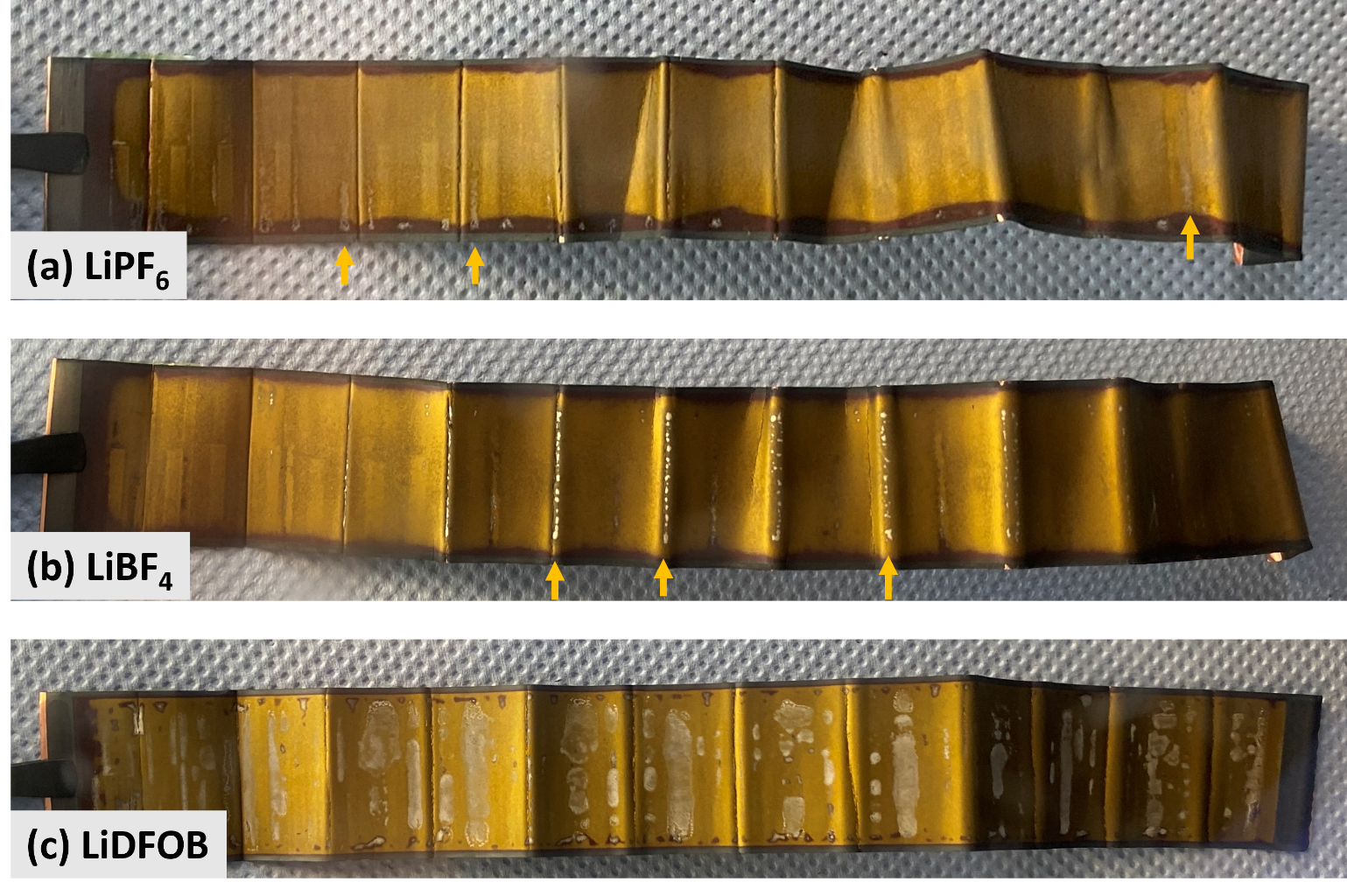


**Figure S15.** Photographs of negative electrodes obtained from cells with electrolytes containing (a) LiPF_6_, (b) LiBF_4_, and (c) LiDFOB at 100% SoC at cycle no. 44.


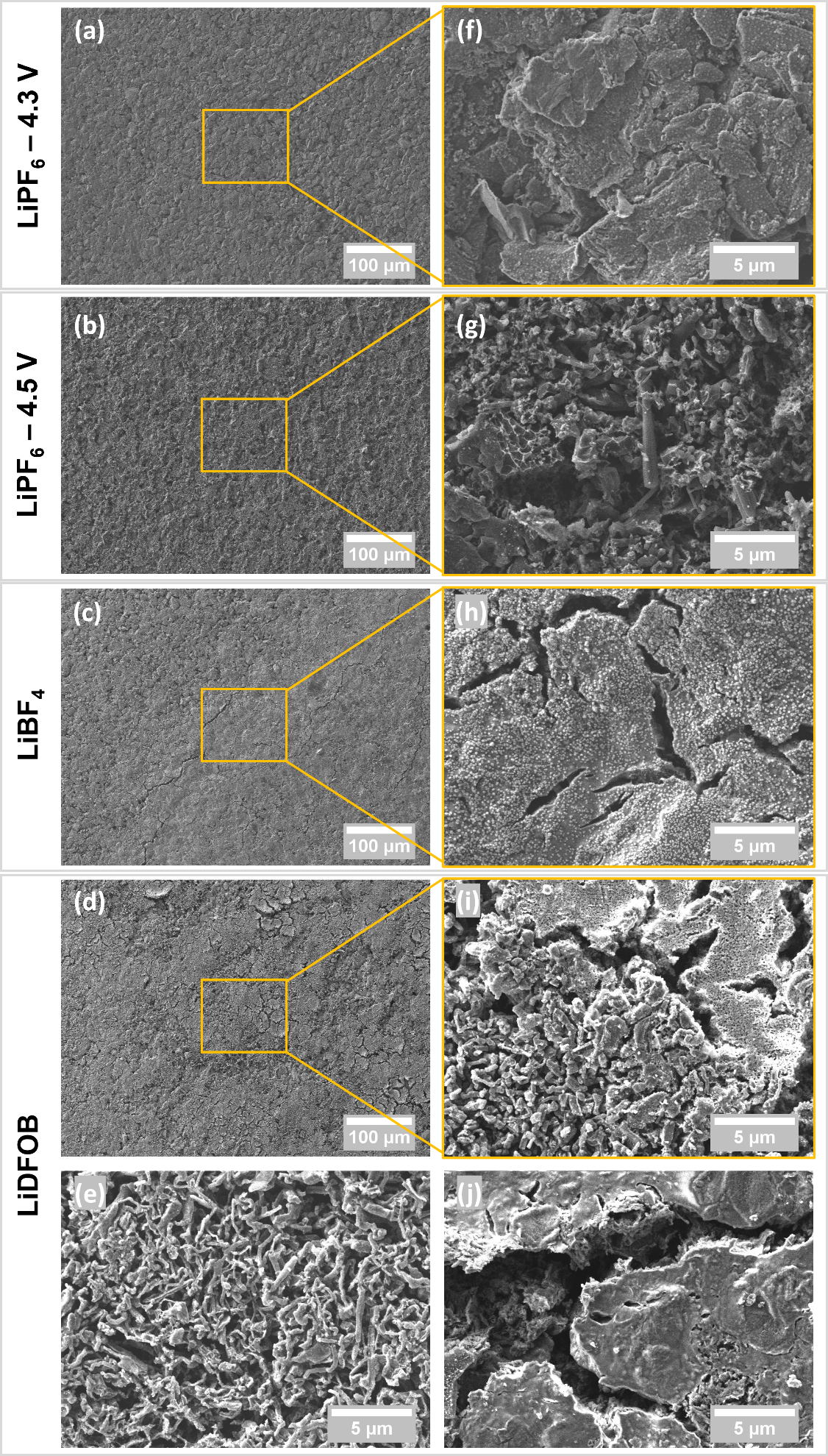


**Figure S16.** (a)‑(d) Low and (e)‑(j) high magnification SEM images of plated lithium on fully discharged negative electrodes obtained from cells with electrolytes containing LiPF_6_, LiBF_4_, and LiDFOB. (e) and (j) are taken from electrode areas not shown in the low magnification SE micrograph (d).

**Reference**

[1] R. Bhattacharyya, B. Key, H. Chen, A. S. Best, A. F. Hollenkamp, C. P. Grey, *Nat. Mater.* **2010**, *9*, 504.

[2] H. J. Chang, N. M. Trease, A. J. Ilott, D. Zeng, L.-S. Du, A. Jerschow, C. P. Grey, *J. Phys. Chem. C* **2015**, *119*, 16443.

[3] Y. Zhu, Y. Li, M. Bettge, D. P. Abraham, *J. Electrochem. Soc.* **2012**, *159*, A2109-A2117.

[4] A. J. Louli, A. Eldesoky, R. Weber, M. Genovese, M. Coon, J. deGooyer, Z. Deng, R. T. White, J. Lee, T. Rodgers, R. Petibon, S. Hy, S. J. H. Cheng, J. R. Dahn, *Nat. Energy* **2020**, *5*, 693.

[5] G. Hernández, A. J. Naylor, Y.-C. Chien, D. Brandell, J. Mindemark, K. Edström, *ACS sustainable chemistry & engineering* **2020**, *8*, 10041.

[6] B. S. Parimalam, B. L. Lucht, *J. Electrochem. Soc.* **2018**, *165*, A251-A255.
